# Association of Hormonal Contraceptive Use With Adverse Health Outcomes: An Umbrella Review of Meta-analyses of Randomized Clinical Trials and Cohort Studies

**DOI:** 10.1001/jamanetworkopen.2021.43730

**Published:** 2022-01-14

**Authors:** Sharmila Brabaharan, Sajesh K. Veettil, Jennifer E. Kaiser, Vrosha Rau Raja Rao, Rujira Wattanayingcharoenchai, Marikannan Maharajan, Putsarat Insin, Pattarawalai Talungchit, Thunyarat Anothaisintawee, Ammarin Thakkinstian, Nathorn Chaiyakunapruk

**Affiliations:** 1 School of Postgraduate Studies, International Medical University, Kuala Lumpur, Malaysia; 2 Department of Pharmacotherapy, College of Pharmacy, University of Utah, Salt Lake City; 3 Department of Obstetrics and Gynecology, University of Utah, Salt Lake City; 4 Department of Pharmacy, Sarikei Public Health Clinic, Sarawak, Malaysia; 5 Department of Obstetrics and Gynecology, Faculty of Medicine, Ramathibodi Hospital, Mahidol University, Bangkok, Thailand; 6 Department of Pharmacy Practice, School of Pharmacy, International Medical University, Kuala Lumpur, Malaysia; 7 Division of Gynecologic Oncology, Department of Obstetrics and Gynecology, Rajavithi Hospital, Bangkok, Thailand; 8 Department of Obstetrics and Gynecology, Faculty of Medicine, Siriraj Hospital, Mahidol University, Bangkok, Thailand; 9 Department of Family Medicine, Faculty of Medicine, Ramathibodi Hospital, Mahidol University, Bangkok, Thailand; 10 Department of Clinical Epidemiology and Biostatistics, Faculty of Medicine, Ramathibodi Hospital, Mahidol University, Bangkok, Thailand; 11 School of Pharmacy, University of Wisconsin–Madison, Madison

## Abstract

**Importance:**

Meta-analyses have reported conflicting data on the safety of hormonal contraception, but the quality of evidence for the associations between hormonal contraceptive use and adverse health outcomes has not been quantified in aggregate.

**Objective:**

To grade the evidence from meta-analyses of randomized clinical trials (RCTs) and cohort studies that assessed the associations between hormonal contraceptive use and adverse health outcomes among women.

**Data Sources:**

MEDLINE, Embase, and the Cochrane Database of Systematic Reviews were searched from database inception to August 2020. Search terms included *hormonal contraception*, *contraceptive agents*, *progesterone*, *desogestrel*, *norethindrone*, *megestrol*, *algestone*, *norprogesterones*, and *levonorgestrel* combined with terms such as *systematic review* or *meta-analysis*.

**Evidence Review:**

The methodological quality of each meta-analysis was graded using the Assessment of Multiple Systematic Reviews, version 2, which rated quality as critically low, low, moderate, or high. The Grading of Recommendation, Assessment, Development and Evaluations approach was used to assess the certainty of evidence in meta-analyses of RCTs, with evidence graded as very low, low, moderate, or high. Evidence of associations from meta-analyses of cohort studies was ranked according to established criteria as nonsignificant, weak, suggestive, highly suggestive, or convincing.

**Results:**

A total of 2996 records were screened; of those, 310 full-text articles were assessed for eligibility, and 58 articles (13 meta-analyses of RCTs and 45 meta-analyses of cohort studies) were selected for evidence synthesis. Sixty associations were described in meta-analyses of RCTs, and 96 associations were described in meta-analyses of cohort studies. Among meta-analyses of RCTs, 14 of the 60 associations were nominally statistically significant (*P* ≤ .05); no associations between hormonal contraceptive use and adverse outcomes were supported by high-quality evidence. The association between the use of a levonorgestrel-releasing intrauterine system and reductions in endometrial polyps associated with tamoxifen use (odds ratio [OR], 0.22; 95% CI, 0.13-0.38) was graded as having high-quality evidence, and this evidence ranking was retained in the subgroup analysis. Among meta-analyses of cohort studies, 40 of the 96 associations were nominally statistically significant; however, no associations between hormonal contraceptive use and adverse outcomes were supported by convincing evidence in the primary and subgroup analyses. The risk of venous thromboembolism among those using vs not using oral contraception (OR, 2.42; 95% CI, 1.76-3.32) was initially supported by highly suggestive evidence, but this evidence was downgraded to weak in the sensitivity analysis.

**Conclusions And Relevance:**

The results of this umbrella review supported preexisting understandings of the risks and benefits associated with hormonal contraceptive use. Overall, the associations between hormonal contraceptive use and cardiovascular risk, cancer risk, and other major adverse health outcomes were not supported by high-quality evidence.

## Key Points

**Question:**

What are the quality and certainty of evidence for an association between hormonal contraceptive use and the risk of adverse health outcomes in meta-analyses of randomized clinical trials and cohort studies?

**Findings:**

In this umbrella review of 58 meta-analyses of randomized clinical trials and cohort studies describing 156 associations between hormonal contraceptive use and adverse health outcomes among women, no associations with adverse outcomes, including cardiovascular and cancer risk, were supported by high-quality evidence. However, the association between the use of a levonorgestrel-releasing intrauterine system and reductions in endometrial polyps associated with tamoxifen use was graded as having high-quality evidence.

**Meaning:**

The results of this umbrella review supported preexisting understandings of the risks and benefits associated with hormonal contraceptive use, suggesting that the associations between hormonal contraceptive use and adverse health outcomes are not supported by high-quality evidence.

## Teaser

This umbrella review assesses evidence from meta-analyses of randomized clinical trials and cohort studies examining the association between hormonal contraceptive use and adverse health outcomes among women.

## Introduction

Hormonal contraceptive use has been increasing worldwide. In 2019, approximately 1.1 billion women of reproductive age were in need of family planning services worldwide, with almost 50% of those women using various hormonal contraceptive methods,^1^ including either estrogen and progesterone or progesterone only. These drugs are available in different dosages and forms, such as tablets, implants, intrauterine systems, intramuscular injections, intravaginal rings, and skin patches.^2^ Overall, when used correctly and consistently, all hormonal contraceptive agents (alone or in combination) have been found to be effective, safe, and reversible forms of contraception.^3^

However, contradictory results have been reported from several meta-analyses^2,4^ on the associations between hormonal contraceptive use and adverse health outcomes. The use of hormonal contraception has been associated with either reduced or increased risk of many adverse health outcomes, including cancer, cardiovascular, fracture, gastrointestinal, and metabolic outcomes.^4^ These risks vary depending on the dose, route, duration of use, and generations and formulations of hormonal contraceptive agents. Because this heterogeneity is present in previous meta-analyses, robust grading of the evidence is needed. To our knowledge, there has been little synthesis of the certainty and quality of the evidence in aggregate across meta-analyses. Umbrella reviews summarize the evidence from multiple meta-analyses on the same topic by assessing the certainty and precision of the associations and the presence of bias, thereby enabling the grading of evidence using well-defined criteria.^5^

This umbrella review aimed to systematically identify relevant meta-analyses of randomized clinical trials (RCTs) and cohort studies of hormonal contraceptive agents, summarize their findings, and assess the certainty of their evidence to provide a comprehensive understanding of the associations between hormonal contraception and adverse health outcomes.

## Methods

The protocol for this study was registered at the International Prospective Register of Systematic Reviews (PROSPERO; registration number: CRD42021231959). The study followed the Preferred Reporting Items for Systematic Reviews and Meta-analyses (PRISMA) reporting guideline^6^ and the Meta-analysis of Observational Studies in Epidemiology (MOOSE) reporting guideline.^7^

### Literature Search and Selection Criteria

A systematic literature search was conducted in MEDLINE, Embase, and the Cochrane Database of Systematic Reviews from database inception until August 2020. Our search strategy combined terms associated with hormonal contraceptive exposure, adverse health outcomes, and meta-analysis. Sample search terms included *hormonal contraception*, *contraceptive agents*, *progesterone*, *desogestrel*, *norethindrone*, *megestrol*, *algestone*, *norprogesterones*, and *levonorgestrel* combined with terms such as *systematic review* or *meta-analysis* (a full list of search terms is available in eTable 1 in the Supplement). No language restriction was applied.

We included meta-analyses of RCTs and cohort studies investigating any association between hormonal contraceptive use and adverse health outcomes, with no restrictions on comparators or populations. We included meta-analyses that reported a higher or lower risk of adverse health outcomes when using hormones for contraception or treatment (eg, polycystic ovarian syndrome [PCOS]). We did not include meta-analyses of nonspecific adverse events, such as abdominal pain and similar gastrointestinal discomforts, acne, leucorrhea, or breast changes. Adverse health outcomes reported in meta-analyses were extracted as defined by the original authors of each meta-analysis. When more than 1 meta-analysis was available for the same research question, we selected the meta-analysis with the largest data set, as previously described elsewhere.^8,9,10^ Detailed inclusion and exclusion criteria and explanations of the selection between overlapping meta-analyses are provided in eTable 2 in the Supplement.

Titles and abstracts of articles were screened by 2 reviewers (S.B. and M.K.) independently. The same 2 reviewers independently assessed the full text of potentially eligible articles. Any discrepancies were resolved by a third reviewer (S.V.) via consensus.

### Data Extraction

Data extraction was independently performed by 2 reviewers (S.B. and V.R.), with any disagreement referred to a third reviewer (S.V.) for resolution. We extracted data at both the meta-analysis and individual study levels (eTable 2 in the Supplement). To grade the methodological quality of each meta-analysis, we used the Assessment of Multiple Systematic Reviews, version 2 (AMSTAR-2) tool, which rated quality as critically low, low, moderate, or high.^11^

### Assessment of Evidence Credibility

For meta-analyses of RCTs, we evaluated the certainty of the evidence for each association using the Grading of Recommendation, Assessment, Development and Evaluations (GRADE) framework, which classified evidence as very low, low, moderate, or high.^12^ For meta-analyses of cohort studies, we applied several criteria in accordance with previous umbrella reviews to grade the quality of evidence.^8,9,13^ Associations from meta-analyses of cohort studies that were nominally significant (ie, *P* ≤ .05) were graded as having convincing (class 1), highly suggestive (class 2), suggestive (class 3), or weak (class 4) evidence based on the amount of evidence, statistical significance, heterogeneity, small-study effect, excess significance bias, prediction interval, and credibility ceiling test (details of classification criteria are provided in Table 1).

**Table 1.** Criteria for Quality of Evidence Classification in Meta-analyses of Cohort Studies

Category	Criteria
Convincing (class 1)	Number of participants >1000 *P* < 10^−6^ *I*^2^ < 50% Largest component study reporting a nominal statistically significant result (*P* < .05) 95% prediction interval excluding the null No small-study effects No excess significance bias Survived 10% credibility ceiling test
Highly suggestive (class 2)	Number of participants >1000 *P* < 10^−6^ Largest study with a statistically significant effect (*P* < .05)
Suggestive (class 3)	Number of participants >1000 *P* < 10^−3^
Weak (class 4)	*P* < .05
Nonsignificant	*P* > .05

### Sensitivity Analysis

We performed a series of sensitivity analyses to determine the robustness of the findings for each association that was initially graded as having high or moderate evidence (for RCTs) or convincing or highly suggestive evidence (for cohort studies). These sensitivity analyses were performed by excluding small studies (<25th percentile) and primary studies with high risk of bias or low-quality evidence.^14^ Another sensitivity analysis was conducted based on the approach used for random-effects meta-analyses. We used the Hartung-Knapp-Sidik-Jonkman approach rather than the DerSimonian and Laird method for associations reported in fewer than 5 studies.^15^

### Statistical Analysis

For each association between hormonal contraceptive exposure and outcome, we extracted effect sizes of individual studies included in each meta-analysis based on study design, and we repeated the meta-analyses separately for RCTs and cohort studies to calculate the pooled effect sizes and 95% CIs using random-effects models.^16^ We assessed heterogeneity using the *I*^2^ statistic.^17^ The evidence for small-study effects was evaluated using the Egger test.^18^

For meta-analyses of cohort studies, we also estimated the 95% prediction interval, which evaluated the uncertainty of effect that would be expected in a new population addressing a similar association.^19^ We also applied excess significance assessment using an χ^2^ test, which evaluated whether the observed number of studies with statistically significant results differed from the expected number of studies with statistically significant results.^20,21^ We also performed a credibility ceiling test using a ceiling of 10% to reflect our level of confidence in the cohort studies.^22,23^ All statistical analyses were conducted using Stata software, version 16.0 (StataCorp LLC). The level of significance was set at 2-sided *P* = .05 for all tests, with the exception of the Egger and excess significance tests, which had significance levels of 2-sided *P* = .10.

## Results

### Study Selection

In total, we screened 2996 records and assessed 310 full-text articles for eligibility. Of those, 102 articles (32.8%) were eligible for preliminary data extraction (eFigure 1 in the Supplement). After the selection criteria for the overlapping meta-analyses were applied (Table 2), 58 articles^24,25,26,27,28,29,30,31,32,33,34,35,36,37,38,39,40,41,42,43,44,45,46,47,48,49,50,51,52,53,54,55,56,57,58,59,60,61,62,63,64,65,66,67,68,69,70,71,72,73,74,75,76,77,78,79,80,81^ were selected for evidence synthesis; of those, 13 articles^24,25,26,27,35,36,42,72,73,74,79,80,81^ were meta-analyses of RCTs, and 45 articles^25,28,29,30,31,33,34,38,40,41,43,44,45,46,47,48,49,50,51,52,53,54,55,56,57,58,59,60,61,62,63,64,65,66,67,68,69,70,71,72,75,76,77,78,79^ were meta-analyses of cohort studies. The list of articles excluded after the selection criteria for the overlapping meta-analyses were applied is provided in eTable 3 in the Supplement.

**Table 2.** Findings of Significant Associations in Meta-analyses of Randomized Clinical Trials

Source	Health outcome	Population	Intervention contraception or treatment	Comparison contraception or treatment	Follow-up duration	Studies, No.	Total participants, No.	Measure	Effect size (95% CI)	*P* value	*I*^2^, %	GRADE overall certainty of evidence	AMSTAR-2
**Increased risk**													
Lethaby et al,^24^ 2015	Weight gain	Women of reproductive age with regular heavy menstruation	LNG-IUS	Ablation	12 mo	2	141	RR	2.60 (1.16 to 5.84) [NR]	2.00 × 10^−2^	0	Moderate	High
	Ovarian cysts	Women of reproductive age >18 y	LNG-IUS	Other medical treatment	3-10 y	3	784	RR	3.05 (1.21 to 7.70) [NR]	1.80 × 10^−2^	0	Very low	High
Amiri et al,^25^ 2017	Fasting insulin level	Women of reproductive age with PCOS	COC (EE, 30 µg, plus DSG, 150 µg)	Non-COC	12 mo	2	76	MD	2.32 (1.15 to 3.49)	1.06 × 10^−4^	3	Moderate	High
	LDL-C level	Women of reproductive age with PCOS	COC (EE, 35 µg, plus CPA, 2 mg)	Non-COC	12 mo	2	33	MD	15.08 (12.74 to 17.43)	1.80 × 10^−36^	0	Low	Low
	LDL-C level	Women of reproductive age with PCOS	COC (EE, 30 µg, plus DRSP, 3 mg)	Non-COC	12 mo	2	79	MD	11.53 (4.73 to 18.34)	1.00 × 10^−3^	0	Very low	Low
	Total cholesterol level	Women of reproductive age with PCOS	COC (EE, 35 µg, plus CPA, 2 mg)	Non-COC	12 mo	2	33	MD	42.20 (17.01 to 67.38)	1.00 × 10^−3^	74.4	Very low	Low
Ralph et al,^26^ 2015	HIV risk	Sub-Saharan African women aged 16-50 y	DMPA	Non-HC	1-2 y	4	15073	HR	1.30 (1.10 to 1.53)	2.00 × 10^−3^	0	Low	Low
**Reduced risk**													
Chin et al,^27^ 2009	Endometrial polyps	Pre- and/or postmenopausal women receiving breast cancer treatment	LNG-IUS	Non–LNG-IUS	2-5 y	4	417	OR	0.22 (0.13 to 0.38) [NR]	5.67 × 10^−8^	0	High	High
Amiri et al,^25^ 2017	FBG level	Women of reproductive age with PCOS	COC (EE, 35 µg, plus CPA, 2 mg)	Non-COC	6 mo	5	132	MD	−2.05 (−2.82 to −1.28)	1.76 × 10^−7^	0	Moderate	Low
	FBG level	Women of reproductive age with PCOS	COC (EE, 30 µg, plus DRSP, 3 mg)	Non-COC	6 mo	4	134	MD	−4.34 (−7.55 to −0.93)	1.20 × 10^−2^	73.5	Very low	Low
	HDL-C level	Women of reproductive age with PCOS	COC (EE, 35 µg, plus CPA, 2 mg)	Non-COC	12 mo	2	33	MD	10.00 (8.41 to 11.59)	5.37 × 10^−35^	0	Low	Low
	HDL-C level	Women of reproductive age with PCOS	COC (EE, 30 µg, plus DRSP, 3 mg)	Non-COC	6 mo	2	91	MD	6.50 (1.91 to 11.09)	1.00 × 10^−3^	47.4	Very low	Low
	HOMA-IR	Women of reproductive age with PCOS	COC (EE, 35 µg, plus CPA, 2 mg)	Non-COC	3 mo	2	42	MD	−0.75 (−1.24 to −0.25)	3.00 × 10^−3^	0	Very low	Low
	Total cholesterol level	Women of reproductive age with PCOS	COC (EE, 35 µg, plus CPA, 2 mg)	Non-COC	6 mo	2	51	MD	−3.67 (−7.26 to −0.07)	4.50 × 10^−3^	0	Very low	Low

Abbreviations: AMSTAR-2, Assessment of Multiple Systematic Reviews, version 2; COC, combined oral contraception; CPA, cyproterone acetate; DMPA, depot medroxyprogesterone acetate; DRSP, drospirenone; DSG, desogestrel; EE, ethinyl estradiol; FBG, fasting blood glucose; HC, hormonal contraception; HDL-C, high-density lipoprotein cholesterol; HIV, human immunodeficiency virus; HOMA-IR, homeostatic model assessment for insulin resistance; HR, hazard ratio; LDL-C, low-density lipoprotein cholesterol; LNG-IUS, levonorgestrel-releasing intrauterine system; MD, mean difference; NR, not reported adjusted or unadjusted effect size; OR, odds ratio; PCOS, polycystic ovarian syndrome; RR, risk ratio.

### Meta-analyses of RCTs

#### Characteristics

Thirteen eligible meta-analyses^24,25,26,27,35,36,42,72,73,74,79,80,81^ described 60 potential associations, including 60 individual study estimates of adverse health outcomes associated with hormonal contraceptive exposure. The median number of RCTs per meta-analysis was 2 (IQR, 2-3), with a median sample of 91 participants (IQR, 75-154 participants) and a follow-up duration ranging from 3 months to 10 years. Additional descriptive characteristics are provided in eTable 4 in the Supplement.

Evaluation of the methodological quality of 13 meta-analyses^24,25,26,27,35,36,42,72,73,74,79,80,81^ using the AMSTAR-2 tool revealed that 8 meta-analyses^24,27,35,36,42,73,74^ (61.5%) were of high quality, 3 meta-analyses^25,26,72^ (23.1%) were of low quality, and 2 meta-analyses^80,81^ (15.4%) were of critically low quality (Table 2; eTable 5 in the Supplement).

#### Findings

Fourteen^24,25,26,27^ of the 60 associations^24,25,26,27,35,36,42,72,73,74,79,80,81^ (23.3%) were nominally statistically significant (*P* ≤ .05) based on random-effects models. A total of 21 associations^25,35^ (35.0%) had high heterogeneity (*I*^2^ > 50%). Small-study effects were found for 3 associations^25,26^ (5.0%). Summaries of all significant and nonsignificant associations are provided in Table 2 and eTable 5 in the Supplement, respectively

Among the 14 statistically significant associations,^24,25,26,27^ 7 associations^24,25,26^ reported an increased risk of adverse health outcomes but no major adverse health outcomes (eg, cardiovascular or cancer-associated outcomes) (Table 2). For treatment purposes, one of the associations^24^ with moderate-quality evidence reported an increased risk of weight gain among women with regular heavy menstrual bleeding who were using a levonorgestrel-releasing intrauterine system (risk ratio [RR], 2.60; 95% CI, 1.16-5.84) compared with women who were not using this system. Another association^25^ with moderate-quality evidence reported an increased fasting insulin level with combined oral contraceptive use (desogestrel and low-dose ethinyl estradiol, 30 µg) for the treatment of PCOS (mean difference, 2.32; 95% CI, 1.15-3.49). No sensitivity analyses were conducted for these 2 associations^24,25^ because of the limited number of studies.

We identified 7 associations^25,27^ between hormonal contraceptive use and reductions in the risk of adverse health outcomes (Table 2). One association^27^ between the use of a levonorgestrel-releasing intrauterine system and a 78% reduction in the risk of endometrial polyps among pre- and postmenopausal women receiving tamoxifen and endometrial surveillance was graded as having high-quality evidence (odds ratio [OR], 0.22; 95% CI, 0.13-0.38). A subgroup analysis of this association including only postmenopausal women retained the same evidence ranking (eTable 6 and eFigure 2 in the Supplement). Another association^25^ between combined oral contraceptive use (cyproterone acetate and ethinyl estradiol, 30 µg) and reductions in fasting blood glucose (FBG) levels among women with PCOS was graded as having moderate-quality evidence (mean difference, −2.05; 95% CI −2.82 to −1.28). This association was upgraded to high-quality evidence after a sensitivity analysis excluding studies with high risk of bias (eTable 6 and eFigure 3 in the Supplement). Among women with PCOS using combined oral contraception, the findings suggested increased high-density lipoprotein levels (mean difference ranging from 6.50 [95% CI, 1.91-11.09] to 10.00 [95% CI, 8.41-11.59]^25^). In addition, women with PCOS experienced increases in low-density lipoprotein levels (mean difference ranging from 11.53 [95% CI, 4.73-18.34] to 15.08 [95% CI, 12.74-17.43]^25^) and total cholesterol levels (mean difference, 42.20; 95% CI, 17.01-67.38^25^). Associations that were initially graded as having evidence of very low to high quality in the primary analysis are provided in eFigure 6 and eFigure 7 in the Supplement, and more details on sensitivity analyses are reported in eTable 6 in the Supplement.

### Meta-analyses of Cohort Studies

#### Characteristics

Forty-five eligible meta-analyses^25,28,29,30,31,33,34,38,40,41,43,44,45,46,47,48,49,50,51,52,53,54,55,56,57,58,59,60,61,62,63,64,65,66,67,68,69,70,71,72,75,76,77,78,79^ described 96 potential associations, including 171 individual study estimates of adverse health outcomes associated with hormonal contraceptive exposure. The median number of studies per meta-analysis was 3 (IQR, 2-5), and the follow-up duration ranged from 3 months to 42 years. The median sample based on 47 associations was 199 participants (IQR, 107-2470 participants). For 29 of 96 associations (30.2%), the number of participants was greater than 1000. Additional descriptive characteristics are provided in Table 3 and eTable 7 in the Supplement, and details of adjustments made for potential confounding variables in the included meta-analyses are available in eTable 7 in the Supplement.

**Table 3.** Findings of Significant Associations in Meta-analyses of Cohort Studies

Source	Adverse health outcome	Population	Exposure contraception or treatment	Nonexposure contraception or treatment	Studies per association, No.	Follow-up duration	Measure	Random effect measure, effect size (95% CI)	Cases, No.	*P* value		*I*^2^, %	Largest study, effect size (95% CI)	Prediction interval (95% CI)	Small-study effect/ excess significance bias	10% Credibility ceiling test, 95% CI	Class of evidence^a^	AMSTAR-2
**Increased risk**																		
Vercellini et al,^46^ 2011	Endometriosis	Women aged 15-90 y or with diagnosis of surgical endometriosis	Past OC	Never OC	5	8-32 y	RR	1.60 (1.40 to 1.82)	>1000	1.32 × 10^−12^	1.6		1.70 (1.47 to 1.96)	1.28 to 2.00	No/no	1.03 to 1.72	1	Moderate
Baratloo et al,^65^ 2014	VTE	Women aged 18-79 y	OC	Never OC	3	6-8 y	OR	2.42 (1.76 to 3.32)	>1000	4.54 × 10^−8^	73.8		2.83 (2.65 to 3.01)	0.07 to 87.33	No/NA^b^	1.07 to 4.45	2^15^	Moderate
Bateson et al,^44^ 2016	VTE	Women aged 10-55 y	COC (low-dose DSG plus EE)	LNG	6	5-10 y	RR	2.05 (1.59 to 2.64)	>1000	2.66 × 10^−8^	81.3		2.83 (2.65 to 3.01)	0.92 to 4.57	Yes/NA^b^	1.21 to 2.76	2	Moderate
Martinez et al,^49^ 2012	VTE	Women of reproductive age <50 y	COC (DSG)	COC (LNG)	8	4-36 y	OR	1.93 (1.31 to 2.85)	1330	9.10 × 10^−4^	89.1		1.13 (0.96 to 1.32)	0.50 to 7.38	No/no	1.06 to 1.44	3	Moderate
Oedingen et al,^69^ 2018	VTE	Women aged 15-49 y	COC (GSD plus EE, 30-40 mg)	COC (LNG)	3	8-9 y	OR	1.45 (1.16 to 1.81)	1772	1.00 × 10^−3^	46.9		1.34 (1.15 to 1.57)	0.16 to 18.17	Yes/no	1.03 to 2.04	3	Critically low
	VTE	Women aged 15-49 y	COC (DSG plus EE, 30-40 mg)	COC (LNG)	3	9-36 y	OR	1.61 (1.28 to 2.02)	696	5.70 × 10^−5^	38.5		1.49 (1.22 to 1.82)	0.18 to 14.44	Yes/no	1.02 to 2.50	4	Critically low
Martinez et al,^49^ 2012	VTE	Women of reproductive age <50 y	COC (DSRP)	LNG	4	5-10 y	OR	1.67 (1.10 to 2.55)	691	2.00 × 10^−2^	82.1		1.35 (1.07 to 7.10)	0.25 to 11.01	No/no	0.91 to 1.68	4	Moderate
	VTE	Women of reproductive age <50 y	COC (GSD)	COC (LNG)	5	4-10 y	OR	1.32 (1.07 to 1.63)	1395	9.00 × 10^−3^	28.2		1.20 (1.04 to 1.39)	0.78 to 2.25	Yes/NA^c^	1.03 to 1.60	4	Moderate
Dragoman et al,^66^ 2018	VTE	Women aged 15-49 y	COC (CPA)	Never OC	2	1-8 y	OR	2.02 (1.31 to 3.11)	>145	1.00 × 10^−3^	3.2		2.11 (1.51 to 2.95)	NA	NA/NA^b^	0.60 to 4.56	4	Moderate
Moorman et al,^54^ 2013	Breast cancer	Women with *BRCA1* or *BRCA2* variant	Ever OC	Never OC	2	14 y (1 cohort)	OR	1.65 (1.32 to 2.06)	1249	8.01 × 10^−6^	44.2		1.84 (1.47 to 2.31)	NA	NA/NA^b^	0.95 to 2.58	3	Moderate
	Breast cancer	Women with *BRCA1* variant	Ever OC	Never OC	2	14 y (1 cohort)	OR	1.59 (1.31 to 1.92)	877	1.70 × 10^−6^	0		1.47 (1.13 to 1.92)	NA	NA/NA^b^	0.96 to 2.51	4	Moderate
	Breast cancer	Women with *BRCA2* variant	Ever OC	Never OC	2	14 y (1 cohort)	OR	1.85 (1.30 to 2.62)	372	6.20 × 10^−4^	0		2.07 (1.34 to 3.20)	NA	NA/NA^b^	0.94 to 2.74	4	Moderate
Asthana et al,^78^ 2020	Cervical cancer	Women aged 25-70 y	Ever OC	Never OC	5	1-42 y	OR	2.18 (1.44 to 3.32	496	2.50 × 10^−4^	51		1.60 (1.10 to 2.30)	0.64 to 7.49	Yes/no	1.12 to 2.67	4	Moderate
	Cervical cancer	Women with aged 25-39 y	Ever OC (>5 y)	Never OC	5	1-42 y	OR	2.83 (1.94 to 4.13)	242	7.08 × 10^−8^	25.4		2.00 (1.30 to 3.00)	1.12 to 7.14	Yes/no	1.32 to 5.65	4	Moderate
	Cervical cancer	Women with HPV	Ever OC	Never OC	4	1-8 y	OR	1.50 (1.07 to 2.11)	336	2.00 × 10^−2^	0		1.70 (0.90 to 3.21)	0.71 to 3.16	Yes/NA^b^	0.90 to 2.04	4	Moderate
Delgado-Rodriguez et al,^33^ 1992	Cancer in situ	Women of reproductive age	Ever OC	Non-HC or never HC	5	3-20 y	OR	1.74 (1.13 to 2.67)	NA	1.00 × 10^−2^	66.9		2.90 (2.00 to 4.10)	0.45 to 6.73	No/NA^b^	0.96 to 1.84	4	Critically low
Xu et al,^62^ 2015	Dry socket	Women with impacted third molar extraction	Current OC	Never OC	16	1-8 d	RR	1.81 (1.34 to 2.43) unadjusted	385	9.60 × 10^−5^	20.3		2.18 (1.64 to 2.89)	0.79 to 4.14	No/NA^c^	1.15 to 2.17	4	High
Liu et al,^28^ 2017	Hypertension	Women aged 18-42 y	OC	Never OC	3	9 mo to 4 y	RR	1.44 (1.06 to 1.97)	1852	2.00 × 10^−3^	76.6		1.20 (1.00 to 1.40)	0.04 to 49.90	No/NA^b^	1.00 to 1.61	4	Critically low
Ortizo et al,^38^ 2017	Inflammatory bowel disease	Women aged 20-89 y	OC	Never OC	3	2-6 y	OR	1.44 (1.02 to 2.02)	189	4.00 × 10^−2^	50.8		1.10 (0.80 to 1.50)	0.04 to 47.12	No/no	0.92 to 1.60	4	Critically low
	Crohn disease	Women aged 20-89 y	OC	Never OC	3	2-6 y	OR	1.51 (1.08 to 2.10)	80	2.00 × 10^−2^	0		1.45 (0.94 to 2.22)	0.17 to 17.06	No/NA^c^	1.01 to 2.29	4	Critically low
Wang et al,^71^ 2019	Ulcerative colitis	Women aged 20-55 y	OC	Never OC	3	3-32 y	OR	1.44 (1.03 to 2.02)	154	3.00 × 10^−2^	41.4		1.18 (0.92 to 1.52)	0.05 to 39.30	No/no	0.98 to 1.58	4	Critically low
Amiri et al,^25^ 2017	Triglyceride level	Women of reproductive age with PCOS	COC (CPA plus EE)	Never OC	3	3 mo	MD	37.84 (22.23 to 53.45)	NA	2.01 × 10^−6^	65.3		48.47 (40.75 to 56.19)	to 391.82 to 467.50	No/NA^b^	1.60 to 50.85	4	Critically low
	Triglyceride level	Women of reproductive age with PCOS	COC (DRSP plus EE)	Never OC	2	6 mo	MD	39.82 (23.43 to 56.22)	NA	1.94 × 10^−6^	0		44.00 (23.74 to 64.25)	NA	NA/NA^b^	to 3.42 to 75.55	4	Critically low
	Triglyceride level	Women of reproductive age with PCOS	COC (CPA plus EE)	Never OC	4	3 mo	MD	13.70 (3.74 to 23.66)	NA	7.00 × 10^−3^	78.4		19.33 (13.73 to 24.93)	NA	NA/NA^b^	to 9.05 to 26.50	4	Critically low
Halperin et al,^47^ 2011	Triglyceride level	Women of reproductive aged 13-44 y	COC	Never OC	6	3-12 mo	MD	0.73 (0.05 to 1.41)	NA	4.00 × 10^−2^	97.7		1.80 (1.67 to 1.94)	−3.77 to 5.23	No/NA^b^	0.08 to 0.53	4	Critically low
Johnston et al,^31^ 1998	Mortality associated with SAH	Women of reproductive age	Ever OC	Never OC	2	7-16 y	OR	4.07 (1.49 to 11.13)	28	6.00 × 10^−3^	0		4.00 (1.30 to 12.90)	NA	NA/NA^b^	1.15 to 2.17	4	Critically low
Xu et al,^61^ 2015	Ischemic stroke	Women aged 15-49 y	Current OC	Never OC	3	8.8-18.2 y	OR	1.67 (1.08 to 2.58)	3402	2.00 × 10^−2^	44.6		1.72 (1.17 to 2.40)	0.02 to 130.40	No/NA^c^	0.86 to 2.19	4	Low
Pérez-López et al,^76^ 2018	Suicide risk	Women aged 25-55 y	OC	Never OC	3	20-39 y	RR	1.36 (1.06 to 1.75)	318	1.00 × 10^−2^	0		1.41 (1.06 to 1.88)	0.27 to 6.84	No/no	0.91 to 1.89	4	Moderate
Shere et al,^59^ 2015	Plasma folate concentration	Women of reproductive age	OC	Never OC	12	3 mo to 8 y	MD	−1.23 (−1.82 to −0.64) [NR]	NA	4.60 × 10^−5^	85.9		0.52 (− 0.83 to −0.21)	−4.56 to 2.10	No/NA^b^	−1.82 to −0.64	4	Low
	RBC folate concentration	Women aged 18-30 y	OC	Never OC	9	3 mo to 4 y	MD	−49.99 (−87.39 to −12.58) [NR]	NA	9.00 × 10^−3^	96.1		−17.27 (−33.10 to −1.44)	−271.19 to 171.21	No/NA^b^	−87.39 to 12.58	4	Low
**Reduced risk**																		
Lan et al,^70^ 2018	Glioma risk	Pre- and postmenopausal women	OC	Never OC	4	7.0-16.4 y	OR	0.75 (0.67 to 0.85) [NR]	1095	2.09 × 10^−6^		0	0.52 (0.64 to 0.87)	0.58 to 0.97	No/NA^c^	0.62 to 0.96	3	Moderate
Luan et al,^64^ 2014	Colorectal adenoma	Women aged 31-90 y	Ever OC	Never OC	8	9-28 y	OR	0.85 (0.78 to 0.92)	4605	6.10 × 10^−5^		20.3	0.92 (0.83 to 1.02)	0.72 to 1.00	No/NA^b^	0.81 to 0.98	4	Critically low
Song et al,^41^ 2019	Colorectal adenoma	Women aged 30-55 y	Ever OC (>5 y)	Never OC	6	10-35 y	OR	0.81 (0.71 to 0.93)	1341	1.50 × 10^−2^		0	1.10 (0.69 to 1.03)	0.67 to 0.99	No/NA^c^	0.72 to 1.02	4	Critically low
Bosetti et al,^45^ 2009	Colorectal adenoma	Women aged 25-59 y	Ever OC (<1 y)	Never OC	6	10-35 y	OR	0.85 (0.74 to 0.99)	1706	4.00 × 10^−2^		0	0.86 (0.70 to 1.06)	0.69 to 1.05	No/NA^c^	0.74 to 1.01	4	Critically low
Amiri et al,^25^ 2017	BMI	Women of reproductive age with PCOS	COC (DRSP, 3 mg, plus EE, 30 µg)	Never OC	3	6 mo	MD	−0.43 (−0.76 to −0.11) [UA]	NA	9.00 × 10^−3^		0	−0.48 (−0.82 to −0.14)	−0.82 to −0.14	No/NA^b^	−0.94 to 0.32	4	Critically low
Liu et al,^40^ 2014	Kidney cancer	Women of reproductive age	Longest duration OC	Never OC	4	12-28 y	SRR	0.80 (0.66 to 0.97)	460	2.00 × 10^−2^		0	0.72 (0.55 to 0.96)	0.53 to 1.21	No/NA^c^	0.67 to 1.06	4	Moderate
Havrilesky et al,^52^ 2013	Ovarian cancer	Women aged 25-71 y	Ever OC	Never OC	7	1-36 y	OR	0.75 (0.61 to 0.92)	1555	6.00 × 10^−3^		74.6	0.74 (0.63 to 0.87)	0.40 to 1.42	No/NA^c^	0.73 to 1.09	4	Moderate
	Ovarian cancer	Women aged 25-71 y	Ever OC (>5 y)	Never OC	5	1-36 y	OR	0.51 (0.27 to 0.97)	1027	4.00 × 10^−2^		78.5	0.56 (0.42 to 0.75)	0.06 to 4.53	No/NA^c^	0.29 to 1.38	4	Moderate
Mantha et al,^50^ 2012	VTE	Women aged 15-53 y	POC	Never OC	3	2-13 y	OR	0.80 (0.64 to 1.00)	1918	5.00 × 10^−2^		0	0.77 (0.61 to 0.98)	0.18 to 3.46	No/NA^c^	0.59 to 1.19	4	Moderate
Vercellini et al,^46^ 2011	Endometriosis	Women aged 15-90 y or with diagnosis of surgical endometriosis	Current OC	Never OC	5	8-32 y	RR	0.57 (0.40 to 0.80)	>1000	1.00 × 10^−3^		43.9	0.80 (0.62 to 1.03)	0.21 to 1.51	Yes/NA^b^	0.53 to 0.93	4	Moderate

Abbreviations: AMSTAR-2, Assessment of Multiple Systematic Reviews, version 2; BMI, body mass index (calculated as weight in kilograms divided by height in meters squared); COC, combined oral contraception; CPA, cyproterone acetate; DRSP, drospirenone; DSG, desogestrel; EE, ethinyl estradiol; GSD, gestodene; HC, hormonal contraception; HPV, human papilloma virus; MD, mean difference; NA, not applicable or not available; NR, not reported adjusted or unadjusted effect size; OC, oral contraception; OR, odds ratio; PCOS, polycystic ovarian syndrome; POC, progesterone-only contraception; RBC, red blood cell; RR, risk ratio; SAH, subarachnoid hemorrhage; SRR, summary risk ratio; UA, unadjusted effect size; VTE, venous thromboembolism.

^a^ Associations that were nominally significant (ie, *P* ≤ .05) were graded as having convincing (class 1), highly suggestive (class 2), suggestive (class 3), or weak (class 4) evidence based on the amount of evidence, statistical significance, heterogeneity, small-study effect, excess significance bias, prediction interval, and credibility ceiling test.

^b^ Not applicable because of nonsignificant effect estimate or unavailable data.

^c^ Not applicable because estimated number was larger than observed, and there was no evidence of excess significance based on assumption made for plausible effect size.

Analysis of the methodological quality of 45 meta-analyses^25,28,29,30,31,33,34,38,40,41,43,44,45,46,47,48,49,50,51,52,53,54,55,56,57,58,59,60,61,62,63,64,65,66,67,68,69,70,71,72,75,76,77,78,79^ using the AMSTAR-2 tool revealed that 6 meta-analyses^41,57,62,66,68,77^ (13.3%) were of high quality, 14 meta-analyses^40,44,46,49,50,52,53,54,63,65,70,75,76,78^ (31.1%) were of moderate quality, 7 meta-analyses^25,48,59,60,61,72,79^ (15.6%) were of low quality, and the remaining 18 meta-analyses^28,29,30,31,33,34,38,43,45,47,51,55,56,58,64,67,69,71^ (40.0%) were of critically low quality (Table 3; eTable 8 in the Supplement).

#### Findings

Forty^25,28,31,33,38,40,43,44,45,46,47,49,50,52,54,59,61,62,64,65,66,69,70,71,76,78^ of the 96 examined associations (41.7%) were nominally statistically significant (*P* ≤ .05), and only 4 associations^44,46,65,78^ (4.2%) reached high significance with a *P* value of 1.00 × 10^−6^ or less (Table 3). Thirty-eight associations^25,28,29,31,33,38,40,41,44,47,48,49,51,52,53,57,59,60,62,64,65,67,69,72,75,77,78^ (39.6%) had high heterogeneity (*I*^2^ > 50%). The 95% prediction interval excluded the null value for only 5 associations^46,64,70,78^ (5.2%). Small-study effects were found for 10 associations^33,44,49,69^ (10.4%), and no excess significance bias was observed for any association. Among 40 statistically significant associations,^25,28,31,33,38,40,43,44,45,46,47,49,50,52,54,59,61,62,64,65,66,69,70,71,76,78^ only 18 associations^25,31,38,44,46,47,49,59,62,64,65,69,70,78^ (45.0%) passed the credibility ceiling test using a ceiling value of 10%. Summaries of all significant and nonsignificant associations are provided in Table 3 and eTable 8 in the Supplement, respectively.

Thirty associations^25,28,31,33,38,44,46,47,49,54,59,61,62,65,66,69,71,76,78^ with an increased risk of adverse health outcomes, including cardiovascular risk, cancer risk, or other major adverse outcomes (such as Crohn disease, ulcerative colitis, and risk of suicide), were statistically significant (Table 3). One of those associations^46^(past oral contraceptive use and increased risk of endometriosis among women undergoing surgery for endometriosis) was supported by class 1 evidence (RR, 1.60; 95% CI, 1.40-1.82). Two associations^44,65^ revealing increased risk of venous thromboembolism (VTE) were graded as having class 2 evidence. One of those associations^65^ was among women using oral contraception compared with women not using oral contraception (OR, 2.42; 95% CI, 1.76-3.32), whereas the other association^44^ was among women using low-dose combined oral contraception (desogestrel and ethinyl estradiol, <50 µg) compared with women using a levonorgestrel-releasing intrauterine system (OR, 2.05; 95% CI, 1.59-2.64). Among women with PCOS receiving combined oral contraception, findings suggested increased triglyceride levels (mean difference ranging from 0.73 [95% CI, 0.05-1.41] to 39.82 [95% CI, 23.43-56.22]^25^). In addition, women with PCOS experienced an increase in total cholesterol levels (mean difference, 13.70; 95% CI, 3.74-23.66^25^).

The association between past oral contraceptive use and the risk of endometriosis^46^ was downgraded from class 1 to class 4 after a subgroup analysis excluding the population with surgically diagnosed endometriosis (eFigure 4 in the Supplement). Of 2 associations^44,65^ initially graded as class 2, neither retained the same evidence ranking after sensitivity analyses (eTable 9 and eFigure 5 in the Supplement). Additional results from the sensitivity and subgroup analyses are provided in eTable 9 in the Supplement.

We also identified 10 significant associations^25,40,41,45,46,50,64,70^ between hormonal contraceptive use and reductions in the risk of adverse health outcomes. However, none of those associations had class 1 or class 2 evidence (Table 3). Associations that were initially graded as having suggestive, highly suggestive, or convincing evidence in the primary analysis are provided in eFigure 8 in the Supplement.

## Discussion

 This umbrella review examined 60 associations reported in 13 meta-analyses of RCTs^24,25,26,27,35,36,42,72,73,74,79,80,81^ and found that none of the 14 statistically significant associations^24,25,26,27^ between hormonal contraceptive use and increases in the risk of adverse outcomes were supported by high-quality evidence in the primary or sensitivity analyses. The association between the use of a levonorgestrel-releasing intrauterine system and reductions in the risk of endometrial polyps among postmenopausal women receiving tamoxifen^27^ was supported by high-quality evidence, and the association with reductions in FBG levels among women with PCOS^25^ was also graded as having high-quality evidence after sensitivity analysis. Another association between the use of cyproterone acetate and ethinyl estradiol, 30 µg, and reductions in FBG levels among women with PCOS^14^ was also graded as having high-quality evidence after sensitivity analysis.

We also assessed 96 associations from 45 meta-analyses of cohort studies.^25,28,29,30,31,33,34,38,40,41,43,44,45,46,47,48,49,50,51,52,53,54,55,56,57,58,59,60,61,62,63,64,65,66,67,68,69,70,71,72,75,76,77,78,79^ We found that none of the 40 statistically significant associations^25,28,31,33,38,40,43,44,45,46,47,49,50,52,54,59,61,62,64,65,66,69,70,71,76,78^ between hormonal contraceptive use and adverse health outcomes were supported by convincing evidence in the primary and subgroup analyses. The risk of VTE among those who used oral contraception compared with those who did not was initially supported by highly suggestive evidence, but the evidence was downgraded to weak in the sensitivity analysis. Overall, the findings of this umbrella review suggested that the associations between hormonal contraceptive use and cardiovascular risk, cancer risk, and other major adverse health outcomes were not supported by high-quality evidence.

### Hormonal Contraception and VTE

All meta-analyses of cohort studies evaluating estrogen-containing hormonal contraceptive agents^44,49,65,66,69^ found that these agents were associated with an approximate 1.5- to 2.5-fold increase in the risk of VTE. None of the meta-analyses had class 1 evidence, but 2 meta-analyses^44,65^ did meet the criteria for class 2 evidence. The remaining meta-analyses^49,66,69^ had class 3 or class 4 evidence; however, the associations between the use of combined oral contraceptive use and VTE risk reported in these meta-analyses were significant, suggesting that concern about VTE risk is warranted. Estrogen increases hepatic production of prothrombotic clotting factors and decreases production of factors that promote clot breakdown, resulting in an increased risk of thrombotic events.^82^ The consequences of the generation of progestin and its ability to promulgate estrogen’s VTE risk have been extensively studied and remain controversial. As observed in 3 of the included meta-analyses,^44,49,69^ the effect size of progestin type was modest and potentially confounded by inconsistent controlling for body mass index (calculated as weight in kilograms divided by height in meters squared) and age, 2 factors known to be associated with VTE risk. Overall, this small variance was unlikely to be clinically meaningful when selecting a type of combined oral contraception.

### Hormonal Contraception and Metabolic Changes

In our umbrella review, metabolic changes were only assessed among women with PCOS.^25^ Therefore, it was difficult to extrapolate these associations to the general population because women with PCOS are typically obese and at risk of metabolic syndrome, insulin resistance, and hyperlipidemia. Estrogen modifies lipid metabolism in the liver and regulates serum lipoprotein levels, resulting in increased high-density lipoprotein and triglyceride levels and decreased low-density lipoprotein and total cholesterol levels.^83^ Progestin type can also have consequences for the extent of lipid profile change induced by exogenous estrogen.^84^ Notably, the types of triglyceride particles increased by ethinyl estradiol have not been associated with increases in the risk of atherosclerotic disease.^85^ Among women with PCOS using combined oral contraception, the findings from our review of meta-analyses of RCTs suggested increases in high-density lipoprotein levels (mean difference ranging from 6.50 to10.00^25^), and the findings from our review of meta-analyses of cohort studies suggested increases in triglyceride levels (mean difference ranging from 0.73^51^ to 39.82^25^). Contrary to previous evidence,^86^ our review found an increase in low-density lipoprotein levels (mean difference ranging from 11.53 to 15.08 in a meta-analysis of RCTs^25^) and total cholesterol levels (mean difference of 42.20 in a meta-analysis of RCTs^25^ and 13.70 in a meta-analysis of cohort studies^25^) among women with PCOS. This difference potentially represents the consequences of different progestin formulations, follow-up durations, and compounding of factors underlying the metabolic changes of PCOS. The quality of evidence was rated class 4 in the cohort studies^25,47^ and ranged from low to very low in the RCTs^25^ for associations between women using combined oral contraception and lipid changes, such as increased total cholesterol and triglyceride levels. In addition, the absolute increase in laboratory values was unlikely to be clinically meaningful.

One meta-analysis of RCTs^25^ had moderate- to high-quality evidence for an association between hormonal contraceptive use and increases in fasting insulin levels as well as decreases in FBG levels among women with PCOS. Although these findings appear to be contradictory, the progestin formulations in the tablets were different; desogestrel was used in the RCT reporting increased fasting insulin levels, and cyproterone acetate was used in the RCT reporting decreased FBG levels. The consequences of combined oral contraception for insulin sensitivity were mainly associated with the progestin component; therefore, the antiandrogenic properties of cyproterone acetate may be associated with decreases in insulin resistance and reductions in circulating androgen levels among women with PCOS, thereby producing the FBG levels observed.^87^ However, the clinical importance of the impact of different progestins was likely to be minimal, and the use of any combined oral contraceptive agent was unlikely to be associated with diabetes among women with PCOS.^88^

### Hormonal Contraception and Breast and Cervical Cancer Risk

In 2 meta-analyses of cohort studies,^54,78^ we found evidence of an approximately 1.5-fold increase in the risk of breast cancer^54^ and an approximately 1.5- to 2.5-fold increase in the risk of cervical cancer^78^ with the use of combined oral contraception, although the evidence was of class 3 and class 4 quality. Notably, the breast cancer associations reported^54^ were among women with *BRCA1* (OMIM 113705) and *BRCA2* (OMIM 600185) variants rather than women in the general population. Individuals with *BRCA1* or *BRCA2* variants have an increased baseline risk of breast cancer, and the additive impact of combined oral contraceptive risks would be magnified.^89,90^ Current and recent use of estrogen-containing hormonal contraception is thought to increase breast cancer risk through a tumor promoter effect rather than an initiator effect on preexisting cancer cells.^91,92^ In addition, any increased risk of breast cancer returns to baseline 10 years after cessation of combined oral contraception.^93^

This umbrella review also found class 4 evidence to suggest an association between combined oral contraceptive use and an increased risk of cervical cancer in a meta-analysis by Asthana et al.^78^ Few studies included in that meta-analysis controlled for human papilloma virus infection status, which is an important factor associated with cervical cancer risk. In the meta-analysis by Asthana et al,^78^ the subgroup analysis of studies including participants with known human papilloma virus infection suggested a modest increase in risk associated with combined oral contraceptive use. However, women using combined oral contraception have been reported to have more sexual partners and higher rates of human papilloma virus infection and to receive a greater number of Papanicolaou tests.^94^ A combination of these factors likely explains the increased risk of cervical cancer among women currently using combined oral contraception. Similar to breast cancer risk, the risk of cervical cancer appears to be time sensitive, with little to no increased risk when combined oral contraception has been used for less than 5 years or has not been used for more than 10 years.^95^

### Benefits Associated With Hormonal Contraception

Our umbrella review suggested several benefits associated with hormonal contraception, including reductions in endometrial polyps in the setting of tamoxifen use^27^ and decreases in the incidence of ovarian and colorectal cancers.^41,45,52,64^ The most significant association that was supported by high-quality evidence was found in a meta-analysis of RCTs^27^ in which the use vs nonuse of a levonorgestrel-releasing intrauterine system was associated with a 78% reduction in the risk of endometrial polyps among those receiving tamoxifen. Tamoxifen, a nonsteroidal selective estrogen receptor modulator, is known to induce the formation of endometrial polyps through its estrogen agonist effects on the endometrial lining.^96^ The use of a levonorgestrel-releasing intrauterine system mitigates this risk through its suppressive action on endometrial proliferation.^27,97^ Of note, controversy exists regarding the use of a levonorgestrel-releasing intrauterine system among patients with progesterone receptor–positive breast cancer because of the potential risk of disease recurrence.^98^ Additional noncontraceptive benefits associated with combined oral contraception include reductions in the risk of ovarian^52^ and colorectal cancers,^41,45,64^ and the results of our review supported these findings. Although these associations^27,49,56,67^ were graded as class 4 in quality, they have also been observed in multiple epidemiological studies.^99,100,101^

Robust grading of previous meta-analyses offers clinicians a tool to better evaluate hormonal contraceptive recommendations for at-risk patients. This grading may assist health care practitioners in the selection of hormonal contraceptive agents for their patients and encourage the consideration of existing individual risk factors, such as VTE, cancer, and cardiovascular risk. The medical eligibility criteria guidelines published by the World Health Organization^102^ and the Centers for Disease Control and Prevention^103,104^ can be used to select appropriate hormonal contraception for specific groups of patients based on these risk factors. Future research would ideally include well-designed RCTs comparing adverse health outcomes among those using vs not using hormonal contraception. However, the feasibility of conducting RCTs large enough to detect many of these adverse outcomes is limited. Rigorous cohort studies may aid in better delineating these risks.^13,84,85,87^

### Limitations

This study has several limitations. The umbrella review focused on existing meta-analyses. We found that some adverse outcomes were not included in these meta-analyses, precluding us from performing a comprehensive evaluation of safety aspects of hormonal contraceptive agents. The quality of all primary studies included in each meta-analysis relied on the assessment reported by the respective meta-analysis. Most systematic reviews and meta-analyses included in our review were focused on estrogen-containing combined oral contraceptive agents. When drawing conclusions about clinical practice from the findings of this umbrella review, it is necessary to be mindful that progestin-only methods (ie, progesterone-only tablets, depot medroxyprogesterone acetate injections, progesterone implants, and levonorgestrel-releasing intrauterine systems) are not represented in a clinically meaningful way. However, the conclusions drawn from the combined oral contraceptive data do have implications for clinical practice.

## Conclusions

In this umbrella review, the associations between hormonal contraceptive use and cardiovascular risk, cancer risk, and other major adverse health outcomes were not supported by high-quality evidence. The findings reinforced preexisting understandings of the risks and benefits associated with hormonal contraceptive agents.

## Notes

Supplement. **eTable 1.** Search Strategy **eTable 2.** Inclusion and Exclusion Criteria **eTable 3.** Excluded Studies After Applying Inclusion Criteria for Overlapping Meta-analyses **eTable 4.** Descriptive Characteristics of Included Meta-analyses of RCTs **eTable 5.** Nonsignificant Associations From Meta-analyses of RCTs **eTable 6.** Sensitivity Analyses of Meta-analysis of RCTs Initially Graded as High or Moderate **eTable 7.** Descriptive Characteristics of Included Meta-analyses of Cohort Studies **eTable 8.** Nonsignificant Associations From Meta-analyses of Cohort Studies **eTable 9.** Sensitivity and Subgroup Analyses of Meta-analysis of Cohort Studies Initially Graded as Convincing or Highly Suggestive **eFigure 1.** PRISMA Flow Diagram **eFigure 2.** Forest Plot of Association Between LNG-IUS and Risk of Endometrial Polyps **eFigure 3.** Forest Plot of Association Between COC (EE, 30 mcg/CPA) and FBG Levels **eFigure 4.** Forest Plot of Association Between Past Oral Contraceptive Users and Endometriosis Risk **eFigure 5.** Forest Plot of Association Between DSG-COC Low Dose and VTE Risk **eFigure 6.** Schematic Network Diagram of Associations in RCTs (Binary Data) **eFigure 7.** Schematic Network Diagram of Associations Between COC Users vs Non-COC Users With PCOS in RCTs (Continuous Data) **eFigure 8.** Schematic Network Diagram of Associations in Cohort Studies
eReferences
