# Supplementary material for: Association of Hormonal Contraceptive Use With Adverse Health Outcomes: An Umbrella Review of Meta-analyses of Randomized Clinical Trials and Cohort Studies
Source: JAMA Netw Open. 2022 Jan 14;5(1):e2143730. doi: 10.1001/jamanetworkopen.2021.43730 (PMC8760614; doi:10.1001/jamanetworkopen.2021.43730)
Supplement: Supplement. — eTable 1. Search Strategy eTable 2. Inclusion and Exclusion Criteria eTable 3. Excluded Studies After Applying Inclusion Criteria for Overlapping Meta-analyses eTable 4. Descriptive Characteristics of Included Meta-analyses of RCTs eTable 5. Nonsignificant Associations From Meta-analyses of RCTs eTable 6. Sensitivity Analyses of Meta-analysis of RCTs Initially Graded as High or Moderate eTable 7. Descriptive Characteristics of Included Meta-analyses of Cohort Studies eTable 8. Nonsignificant Associations From Meta-analyses of Cohort Studies eTable 9. Sensitivity and Subgroup Analyses of Meta-analysis of Cohort Studies Initially Graded as Convincing or Highly Suggestive eFigure 1. PRISMA Flow Diagram eFigure 2. Forest Plot of Association Between LNG-IUS and Risk of Endometrial Polyps eFigure 3. Forest Plot of Association Between COC (EE, 30 mcg/CPA) and FBG Levels eFigure 4. Forest Plot of Association Between Past Oral Contraceptive Users and Endometriosis Risk eFigure 5. Forest Plot of Association Between DSG-COC Low Dose and VTE Risk eFigure 6. Schematic Network Diagram of Associations in RCTs (Binary Data) eFigure 7. Schematic Network Diagram of Associations Between COC Users vs Non-COC Users With PCOS in RCTs (Continuous Data) eFigure 8. Schematic Network Diagram of Associations in Cohort Studies eReferences [file jamanetwopen-e2143730-s001.pdf]

Supplementary Online Content

Brabaharan S, Veettil SK, Kaiser JE, et al. Association of hormonal contraceptive use with adverse health outcomes: an umbrella review of meta-analyses of randomized clinical trials and cohort studies. *JAMA Netw Open*. 2022;5(1):e2143730. doi:10.1001/jamanetworkopen.2021.43730

eTable 1. Search Strategy

eTable 2. Inclusion and Exclusion Criteria

eTable 3. Excluded Studies After Applying Inclusion Criteria for Overlapping Meta-analyses

eTable 4. Descriptive Characteristics of Included Meta-analyses of RCTs

eTable 5. Nonsignificant Associations From Meta-analyses of RCTs

eTable 6. Sensitivity Analyses of Meta-analysis of RCTs Initially Graded as High or Moderate

eTable 7. Descriptive Characteristics of Included Meta-analyses of Cohort Studies

eTable 8. Nonsignificant Associations From Meta-analyses of Cohort Studies

eTable 9. Sensitivity and Subgroup Analyses of Meta-analysis of Cohort Studies Initially Graded as Convincing or Highly Suggestive

eFigure 1. PRISMA Flow Diagram

eFigure 2. Forest Plot of Association Between LNG-IUS and Risk of Endometrial Polyps

eFigure 3. Forest Plot of Association Between COC (EE, 30 mcg/CPA) and FBG Levels

eFigure 4. Forest Plot of Association Between Past Oral Contraceptive Users and Endometriosis Risk

eFigure 5. Forest Plot of Association Between DSG-COC Low Dose and VTE Risk

eFigure 6. Schematic Network Diagram of Associations in RCTs (Binary Data)

eFigure 7. Schematic Network Diagram of Associations Between COC Users vs Non-COC Users With PCOS in RCTs (Continuous Data)

eFigure 8. Schematic Network Diagram of Associations in Cohort Studies

eReferences

This supplementary material has been provided by the authors to give readers additional information about their work.

© 2022 Brabaharan S et al. *JAMA Network Open*.

eTable 1. Search Strategy

Databases

1) Medline
2) Embase
3) Cochrane Database of Systematic Review (CDSR)

No	Search term
	**CONTRACEPTIVE**
1)	exp contraception/
2)	exp postcoital/
3)	exp hormonal contraception/
4)	exp long-acting reversible contraception/
5)	exp Contraceptives, Oral/
6)	exp Contraceptive Agents, Female/
7)	exp contraceptives, oral, sequential/
8)	exp contraceptives, postcoital/
9)	exp progesterone/
10)	exp Hydroxyprogesterones/
11)	Hydroxyprogesterone.ti,ab.
12)	exp 20-alpha-dihydroprogesterone/
13)	exp desogestrel$
14)	norethindrone$ Acetate.ti,ab
15)	(megestrol$ Acetate or Megestrol).ti,ab.
16)	norethynodrel.ti,ab.
17)	exp algestone/
18)	algestone$.ti,ab
19)	norprogesterones$.ti,ab.
20)	exp Levonorgestrel/
21)	mirena.ti,ab.
22)	norethisteron$.ti,ab.
23)	medrogestone$.ti,ab.
24)	exp dydrogesterone/
25)	dydrogesterone,ti.ab
26)	melengestrol adj2 acetate.ti,ab
27)	exp desogestrel/
28)	desogestrel.ti,ab.

© 2022 Brabaharan S et al. *JAMA Network Open*.

29)	exp norgestrel/
30)	norgestrel,ti.ab
31)	exp norethindrone/
32)	norethindrone$.ti,ab.
33)	progestat$.ti,ab.
34)	medroxyprogest$ acetate.ti,ab.
35)	intrauterine adj2 contracep$.ti,ab
36)	(levonorgestrel adj2 intrauterine device).ti,ab
37)	nuvaring.ti,ab.
38)	((depo adj2 provera) or depo provera$).ti,ab.
39)	(Depo-medroxyprogest$ or (Depo$ adj2 medroxyprogest$)).ti,ab.
40)	exp Ethynodiol Diacetate/
41)	ethynodiol diacetate.ti,ab.
42)	exp Gestonorone Caproate/
43)	gestonorone caproate.ti,ab
44)	exp lynestrenol/
45)	lynestrenol.ti,ab.
46)	exp ethylestrenol/
47)	ethylestrenol.ti,ab.
48)	exp norethandrolone/
49)	Norethandrolone.ti,ab.
50)	((progesterone adj2 capsule$) or (progest$ adj2 capsule$)).mp.
51)	(progest$ adj2 implant).ti,ab.
52)	(implanon$ or Nexplanon$ or jadelle$ or norplant$ or uniplant$ or sino-implant$).ti,ab.
53)	exp contraceptives, oral, combined/
54)	exp Estrogens/
55)	exp "Estrogens, Conjugated (USP)"/
56)	exp Estradiol/
57)	(ethinyl estradiol and norgestrel).ti,ab
58)	estradiol$ norgestrel.ti,ab
59)	((ethinyl adj2 estradiol) and norgestimate).ti,ab.
60)	(ethinyl adj2 estradiol and gestodene).ti,ab.
61)	(chlormadinone acetate and ethinylestradiol).ti,ab.
62)	(chlormadinone acetate and mestranol).ti,ab.

© 2022 Brabaharan S et al. *JAMA Network Open*.

63)	(desogestrel and ethinylestradiol).ti,ab.
64)	(dienogest and ethinylestradiol).ti,ab
65)	(drospirenone and ethinylestradiol).ti,ab
66)	(ethinylestradiol and ethisterone).ti,ab
67)	(estradiol cypionate and medroxyprogest$ acetate).ti,ab
68)	(ethinylestradiol and levonorgestrel).ti,ab
69)	(ethinylestradiol and megestrol acetate).ti,ab
70)	(ethinylestradiol and norethisterone).ti,ab
71)	(mestranol and norethisterone).ti,ab
72)	triphasic contracept$ agent$.ti,ab.
73)	(chlormadinone acetate and dienogest).ti,ab
74)	(ethinyl estradiol and cyproterone acetate).ti,ab
75)	(ethinyl estradiol and drospirenone).ti,ab.

AND

	**SYTEMATIC REVIEW AND META ANALYSIS**
1)	exp "Systematic Review"/
2)	(systematic adj2 review$).ti,ab.
3)	"systematic review".ti,ab.
4)	exp Meta-Analysis as Topic/
5)	exp Meta-Analysis/
6)	meta-analys$.ti,ab.
7)	(meta adj1 analys$).ti,ab.

© 2022 Brabaharan S et al. *JAMA Network Open*.

eTable 2. Inclusion and Exclusion Criteria

**Selection process**	
**Review question**	Is hormonal contraceptive (HC) use associated with adverse health outcomes?<br>How reliable is the evidence behind this association in published meta-analyses?
**Objective**	To conduct an umbrella review of meta-analyses to gain a systematic, comprehensive overview of the existing evidence from meta-analyses of cohort studies and RCTs on HCs use and adverse health outcomes and to assess its strength and validity.
**Inclusion criteria**	Meta-analyses of cohort studies and meta-analyses of RCTs.<br>**PICO**: participants/population: Pre-and post-menopausal women and women of reproductive age prescribed with HC for contraception and treatment: intervention(s), exposure(s): HCs (Oral contraceptives, injectable contraceptives, implants, patches, intravaginal rings, intrauterine system) for any condition at any dose; comparator(s)/control: no limitation will be applied for comparators; outcome: any adverse health outcome associated with exposure to HCs as defined by the original authors.
**Exclusion criteria**	We have excluded studies which have assessed non-contraceptive hormones such as tocolytic agents, hormonal replacement therapy for post- menopausal women, appetite stimulant, etc. We excluded 1) meta-analyses of studies with other study designs (e.g., cross-sectional, nested case-control and case-control studies); 2) pooled analyses of a non-systematic selection of observational studies, non-randomized trials and non-systematic reviews; and 3) meta-analyses that provided insufficient or inadequate data for quantitative synthesis.
**Search**	Search strategy used a combination of terms related to HC, to adverse health outcomes, and to meta-analysis with no limitations to age. Manual searches of the reference list of eligible articles were conducted to identify additional studies that may not have been retrieved through search strategies. No language restriction was applied for the selection process.
**Data extraction**	
**Preliminary data extraction (Phase I)**	For each eligible meta-analysis, we extracted the following data independently on to a Microsoft Excel spreadsheet (XP professional edition; Microsoft Corp): first author, year of publication, design, and the number of included studies, specific population under investigation, indication of intervention, the number of cases and participants in each arm, the total number of cases and participants, details of the treatment regimen, the control conditions, the adverse outcome (as defined by authors), the stated summary meta-analytic estimates and their corresponding 95% confidence interval (95% CI).

© 2022 Brabaharan S et al. *JAMA Network Open*.

	Based on this information, we generated all possible associations. Then we applied the following criteria to prioritize meta-analysis for each association:<br><br>**Selection between overlapping meta-analyses** [1–3]<br><br>Association from meta-analyses from the largest data set is selected whenever there is more than one meta-analysis reported the same adverse health outcome.[4–6] An association by meta-analysis with the largest number of primary studies in RCT was prioritised. If more than one published meta-analysis on the same adverse health outcome included an equal number of studies, the one with the largest number of cases will be chosen. If more than one published meta-analysis fulfilled both criteria, the one with more available information on primary studies will be chosen. For meta-analyses of cohort studies, a similar approach was adhered if more than one published meta-analysis on the same outcome was identified.
**Data extraction for evidence synthesis (Phase II data synthesis)**	Once we have selected a meta-analysis based on the criteria, the following data were extracted independently onto a separate Microsoft Excel spreadsheet (XP professional edition; Microsoft Corp) for cohort studies and RCTs:<br><br>Meta-analysis level: First author, year of publication, design, and the number of included studies, specific population under investigation, indication of intervention, the number of cases and participants in each arm, total number of cases and participants, details of the treatment regimen, control conditions, follow-up period (mean or median), adverse outcome (as defined by authors), stated summary meta-analytic estimates and their corresponding 95% CI, confounding factors, evidence grading (if reported), and quality score according to AMSTAR 2.<br><br>Individual study level: First author, year of publication, design (e.g.: retrospective cohort or prospective cohort, the number of cases and participants in each arm, total number of cases and participants, quality assessment (Newcastle-Ottawa Scale (NOS)),[7] if reported, stated summary meta-analytic estimates and their corresponding 95%CI.<br><br>Meta-analyses investigating several outcomes were recorded separately in the data extraction sheets. We excluded other study designs during this stage. We performed meta-analysis using random-effects models based on the data from only RCTs or cohort studies separately. We presented summary meta-analytic estimates as follow: risk ratio (RR), odds ratio (OR), hazard ratio (HR), standardized mean difference or mean difference (SMD or MD), or weighted mean difference (WMD).
**Quality assessment**	To grade the methodological quality of each meta-analysis (high, moderate, low, or critically low), we used the revised AMSTAR 2 tool, a 16-item instrument to assess the methodological quality of systematic reviews of randomized and non-randomized studies.[8] The risk of bias (ROB) within each primary study in RCT was assessed using Cochrane Risk of Bias Tool Version 2,[9] whereas risk of bias in primary cohort studies was reported as it was originally stated by the author.

© 2022 Brabaharan S et al. *JAMA Network Open*.

eTable 3. Excluded Studies After Applying Inclusion Criteria for Overlapping Meta-analyses

Reason for exclusion of meta-analysis: either not with the largest number of primary cohort studies or the largest number of cases.

No	*Title of Article*	*Author (Ref)*
1.	Efficacy and side-effects profile of the ethinylestradiol and etonogestrel contraceptive vaginal ring: a systematic review and meta-analysis. Eur J Contracept Reprod Health Care. 2017;22(2):131–46.	Lopez-Picado A 2017[10]
2.	Intrauterine Device Use and Cervical Cancer Risk: A Systematic Review and Meta-analysis. Obstet Gynecol. 2017;130(6):1226–36.	Cortessis VK 2017[11]
3.	Is oral contraceptive use associated with an increased risk of cervical cancer? An evidence-based meta-analysis. J Obstet Gynaecol Res. 2017;43(5):913–22.	Peng Y 2017[12]
4.	Is the oral contraceptive or hormone replacement therapy a risk factor for cholelithiasis: A systematic review and meta-analysis. . Medicine (Baltimore). 2017;96(14):e6556.	Wang S 2017[13]
5.	A pooled analysis of case-control studies of thyroid cancer. III. Oral contraceptives, menopausal replacement therapy and other female hormones. Cancer Causes Control. 1999;10(2):157–66.	La Vecchia C 1999[14]
6.	Do oral contraceptive agents affect the risk of breast cancer? A meta-analysis of the case-control reports. J Am Board Fam Pract. 1993;6(2):123–35.	Hawley W 1992[15]
7.	Contraception and the risk of ectopic pregnancy: A meta-analysis. Contraception. 1995 Dec 1;52(6):337–41.	Mol BWJ 1995[16]
8.	Oral contraceptives and breast cancer. Review and meta-analysis. Cancer. 1990;66(11):2253–63.	Romieu I 1990[17]
9.	Oral contraceptives and the risk of rheumatoid arthritis: a meta-analysis of a conflicting literature. Br J Rheumatol. 1989;28 Suppl 1(b1t, 8302415):13–23.	Romieu I 1989[18]
10.	Oral contraceptives and venous thromboembolism: a quantitative discussion of the uncertainties. J Intern Med. 1995;238(1):31–7	Koster T 1995[19]

© 2022 Brabaharan S et al. *JAMA Network Open*.

11.	The protective effect of the oral contraceptive pill on rheumatoid arthritis: an overview of the analytic epidemiological studies using meta-analysis. J Clin Epidemiol. 1990;43(11):1221–30.	Spector TD 1990[20]
12.	A pooled analysis of 10 case-control studies of melanoma and oral contraceptive use. Br J Cancer. 2002;86(7):1085–92.	Karagas MR 2002 [21]
13.	Oral contraceptives and colorectal cancer risk: a meta-analysis. Br J Cancer. 2001;84(5):722–7.	Fernandez E 2001[22]
14	Risk of venous thromboembolism from oral contraceptives containing gestodene and desogestrel versus levonorgestrel: a meta-analysis and formal sensitivity analysis. Contraception. 2001;64(2):125–33	Hennessy S 2001 [23]
15	Oral contraceptive use and breast cancer risk: a meta-analysis of variations with age at diagnosis, parity and total duration of oral contraceptive use. Br J Obstet Gynaecol. 1992;99(3):239–46.	Rushton L 1992 [24]
16.	Hormonal contraceptives and cerebral venous thrombosis risk: a systematic review and meta-analysis. Front Neurol. 2015;6(101546899):7	Amoozegar F 2015 [25]
17.	Oral contraceptives<br>and risk of ovarian and breast cancers in BRCA mutation carriers: a meta-analysis. Expert Rev Anticancer Ther 2011;11(8):1197–1207.	Cibula D 2011[26]
18.	Depot medroxyprogesterone acetate and breast cancer. A pooled analysis of the World Health Organization and New Zealand studies. JAMA. 1995;273(10):799–804.	Skegg DC 1995 [27]
19.	Association between the current use of low-dose oral contraceptives and cardiovascular arterial disease: a meta-analysis. J Clin Endocrinol Metab. 2005;90(7):3863–70.	Baillargeon-J 2005[28]
20.	Combination injectable contraceptives for contraception. Cochrane Database Syst Rev. 2005;(3):CD004568.	Gallo MF 2005[29]
21.	Thrombophilic abnormalities, oral contraceptives, and risk of cerebral vein thrombosis: a meta-analysis. Blood. 2006;107(7):2766–73.	Dentali F 2006[30]

© 2022 Brabaharan S et al. *JAMA Network Open*.

22	Oral contraceptive use as a risk factor for premenopausal breast cancer: a meta-analysis. Mayo Clin Proc. 2006;81(10):1290–302.	Kahlenborn C 2006 [31]
23	Oral contraceptives containing drospirenone for premenstrual syndrome. Cochrane Database Syst Rev. 2012;(2):CD006586.	Lopez LM 2012[32]
24.	The risk of oral contraceptives in the etiology of inflammatory bowel disease: a meta-analysis. Am J Gastroenterol. 2008;103(9):2394–400.	Cornish JA 2008[33]
25.	Progestogen-only contraceptives and the risk of stroke: a meta-analysis. Stroke. 2009;40(4):1059–62.	Chakhtoura Z 2009[34]
26.	Oral contraceptive use and breast or ovarian cancer risk in BRCA1/2 carriers: a meta-analysis. Eur J Cancer. 2010;46(12):2275–84	Iodice S 2010[35]
27.	Progestogen-only contraceptives and the risk of acute myocardial infarction: a meta-analysis. J Clin Endocrinol Metab. 2011;96(4):1169–74.	Chakhtoura Z 2011[36]
29.	Oral contraceptives and venous thromboembolism: a systematic review and meta-analysis. Drug Saf. 2012;35(3):191–205.	Manzoli L 2012[37]
29.	Types of progestogens in combined oral contraception: effectiveness and side-effects. Cochrane Database Syst Rev. 2011;(5):CD004861.	Lawrie TA 2011[38]
30.	Different combined oral contraceptives and the risk of venous thrombosis: systematic review and network meta-analysis. BMJ [Internet]. 2013 Sep 12 [cited 2020 Jul 26];347.	Stegeman BH 2013[39]
31.	Long-term effects of levonorgestrel-releasing intrauterine system on tamoxifen-treated breast cancer patients: a meta-analysis. Int J Clin Exp Pathol. 2014;7(10):6419–29.	Fu Y 2014[40]
32.	The Role of Oral Contraceptive Pills on Increased Risk of Breast Cancer in Iranian Populations: A Meta-analysis. J cancer prev. 2016;21(4):294–301.	Soroush A 2016[41]
33.	Association between oral contraceptive use as a risk factor and triple-negative breast cancer: A systematic review and meta-analysis. Mol clin oncol. 2017;7(1):76–80.	Li L 2017[42]

© 2022 Brabaharan S et al. *JAMA Network Open*.

34.	Combined oral contraceptives, thrombophilia and the risk of venous thromboembolism: a systematic review and meta-analysis. J Thromb Haemost. 2016;14(7):1393–403.	Van Vlijmen 2016[43]
35.	Effect of age at first use of oral contraceptives on breast cancer risk: An updated meta-analysis. Medicine (Baltimore). 2019;98(36):e15719.	Ji LW 2019[44]
36.	Efficacy of levonorgestrel releasing intrauterine system as a postoperative maintenance therapy of endometriosis: A meta-analysis. Eur J Obstet Gynecol Reprod Biol. 2018;231(e4l, 0375672):85–92.	Song SY 2018[45]
37.	Combined oral contraceptives: the risk of myocardial infarction and ischemic stroke. Cochrane Database Syst Rev. 2015 Aug 27;(8):CD011054.	Roach REJ 2015[46]
38.	Safety of hormonal replacement therapy and oral contraceptives in systemic lupus erythematosus: a systematic review and meta-analysis. PLoS ONE. 2014;9(8):e104303.	Rojas Villarraga A 2014[47]
39.	The effectiveness of metformin, oral contraceptives, and lifestyle modification in improving the metabolism of overweight women with polycystic ovary syndrome: a network meta-analysis. Endocrine. 2019;64(2):220–32.	Wang A 2019[48]
40.	Second- and third-generation oral contraceptives and myocardial infarction: Systematic review and meta-analysis. Clin Invest Ginecol Obstet. 2016;43(4):174–8.	Rojas RF 2016[49]
41.	A Systematic Review and Meta-analysis of the Adverse Effects of Levonorgestrel Emergency Oral Contraceptive. Clin Drug Invest. 2020;40(5):395–420.	Leelakanok N 2020[50]
42.	Modifiers of cancer risk in BRCA1 and BRCA2 mutation carriers: systematic review and meta-analysis. J Natl Cancer Inst;2014. 106(6):dju091	Friebel TM 2014[51]
43.	Meta-analysis of oral contraceptive use and risks of all-cause and cause-specific death. Int J Gynaecol Obstet. 2015;131(3):228–33.	Zhong G-C 2015[52]

© 2022 Brabaharan S et al. *JAMA Network Open*.

44.	The effects of Diane-35 and metformin in treatment of polycystic ovary syndrome: An updated systematic review. Gynaecological Endrocrinology. 2008;24(10);590-600.	Zhang J 2008[53]

© 2022 Brabaharan S et al. *JAMA Network Open*.

eTable 4. Descriptive Characteristics of Included Meta-analyses of RCTs

Source	Adverse health outcome	Population	Exposed/ Unexposed	No of studies	Exposed Cases/total number	Unexposed Cases/total number	Length of follow-up	AMSTAR-2 quality
Significant association								
Lethaby A [54]	Weight gain	Women at reproductive age with regular heavy menstrual	LNG-IUS/Non-user	2	18/70	7/71	12 months	High
Chin J[55]	Endometrial polyps	Post/pre + post-menopausal women with adjuvant tamoxifen for breast cancer	LNG-IUS/Non-users	2	2/113	13/110	1 year	High
Chin J[55]	Endometrial polyps	Post/pre + post-menopausal women with adjuvant tamoxifen for breast cancer	LNG-IUS/Non-users	4	10/206	48/211	2-5 years	High
Amiri M[56]	Fasting insulin 12 months	Women of reproductive age with PCOS	COC (EE 30 mcg + DSG 150 mcg)/Non-users	2	NA	NA	12 months	Low
Amiri M[56]	FBG 6 months	Women of reproductive age with PCOS	COC (EE 30 mcg + DRSP 3 mg)/Non-users	4	NA	NA	6 months	Low
Amiri M[56]	FBG 6 months	Women of reproductive age with PCOS	COC (EE 35 mcg + CPA 2 mg) /Non-users	5	NA	NA	6 months	Low
Amiri M[56]	HOMA IR 3 months	Women of reproductive age with PCOS	COC (EE 35 mcg + CPA 2 mg) /Non-users	2	NA	NA	3 months	Low
Ralph L[(58)]	HIV risk	Sub Saharan African women aged 16-50 years	DMPA/Non-HC user	4	NA/5336	NA/9737	2-2.8 years	Low

© 2022 Brabaharan S et al. *JAMA Network Open*.

Amiri M[56]	TC	Women of reproductive age with PCOS	EE 35 mcg + CPA 2 mg/Non-users	2	NA	NA	6 months	Low
Amiri M[56]	TC	Women of reproductive age with PCOS	EE 35 mcg + CPA 2 mg/Non-users	2	NA	NA	12 months	Low
Amiri M[56]	LDL-C	Women of reproductive age with PCOS	EE 35 mcg + CPA 2 mg/Non-users	2	NA	NA	12 months	Low
Amiri M[56]	HDL-C	Women of reproductive age with PCOS	EE 35 mcg + CPA 2 mg/Non-users	2	NA	NA	12 months	Low
Amiri M[56]	LDL-C	Women of reproductive age with PCOS	EE 35mcg + DRSP 3 mg/Non-users	2	NA	NA	12 months	Low
Amiri M[56]	HDL-C	Women of reproductive age with PCOS	EE 35mcg + DRSP 3 mg/Non-users	2	NA	NA	6 months	Low
Lethaby A[54]	Ovarian cyst	Women of reproductive age >18 years old	LNG-IUS/Other medical treatment	3	17/390	5/394	3-10 years	High
Non-Significant association								
Amiri M[56]	BMI change	Women of reproductive age with PCOS	EE 35 mcg + CPA 2 mg/Non-users	5	NA	NA	3 months	Low
Amiri M[56]	BMI change	Women of reproductive age with PCOS	EE 35 mcg + CPA 2 mg/Non-users	5	NA	NA	6 months	Low
Amiri M[56]	BMI change	Women of reproductive age with PCOS	EE 35 mcg + CPA 2 mg/Non-users	3	NA	NA	12 months	Low
Amiri M[56]	BMI change	Women of reproductive age with PCOS	EE 35 mcg + DRSP 3 mg/Non-users	3	NA	NA	6 months	Low

© 2022 Brabaharan S et al. *JAMA Network Open*.

Amiri M[56]	BMI change	Women of reproductive age with PCOS	EE 35 mcg + DRSP 3 mg/Non-users	3	NA	NA	12 months	Low
Lopez LM[57]	Weight gain	Women at reproductive age >18 years	Vaginal ring etonogestrel 120 mcg/EE 15 mcg/ COC (LNG 150 mcg-EE 30 mcg)	2	11/544	12/588	11-13 months	High
Draper[58]	Weight gain	Women at reproductive age	DMPA/NET-EN	2	NA/871	NA/492	12 months	High
Gallo MF[59]	Depression	Women aged 18-39 years	EE 20 mcg + GSD 75 mcg/ EE 30 mcg + GSD 75 mcg	2	14/459	4/258	12-13 months	High
French R[60]	Ectopic pregnancy	Women aged 18-40 years	LNG-IUS 20/Non-hormonal IUD	2	1/1985	1/1094	1 year	High
French R[60]	Ectopic pregnancy	Women of reproductive age and mixed parity	Progestasert®	2	3/358	0/350	1 year	High
Chin J[55]	Endometrial fibroids	Post/pre + post-menopausal women with adjuvant tamoxifen for breast cancer	LNG-IUS/Non-users	3	4/152	9/162	1-2 years	High
Lopez LM[61]	FBG at cycle 6	Reproductive aged women without diabetes	EE 30/DSG/EE 30/GSD	2	NA/25	NA/32	6 months	Critically low
Lopez LM[61]	FBG at cycle 6	Reproductive aged women without diabetes	EE 30/DSG/ EE 30/LNG	2	NA/33	NA/39	6 months	Critically low
Lopez LM[61]	FBG at cycle 6	Reproductive aged women without diabetes	EE 35/Norethindrone/ EE 30-40/LNG	2	NA/72	NA/72	6 months	Critically low
Lopez LM[61]	FBG at cycle 12	Reproductive aged women without diabetes	EE 30/DSG/ EE 30/LNG	2	NA/27	NA/35	12 months	Critically low

© 2022 Brabaharan S et al. *JAMA Network Open*.

Lopez LM[61]	Glucose area under curve at cycle 6	Reproductive aged women without diabetes	EE 35/Norethindrone/ EE 30-40/LNG	2	NA/72	NA/72	6 months	Critically low
Amiri M[56]	FBG 6 months	Women with PCOS	OC/Non-users	2	NA	NA	3-24 months	Low
Amiri M[56]	FBG 12 months	Women with PCOS	COC (EE 30 mcg + DSG 150 mcg)/Non-users	2	NA	NA	12 months	Low
Amiri M[56]	Fasting insulin 6 months	Women with PCOS	COC (EE 30 mcg + DSG 150 mcg)/Non-users	2	NA	NA	6 months	Low
Amiri M[56]	HOMA-IR	Women with PCOS	COC (EE 30 mcg + DSG 150 mcg)/Non-users	2	NA	NA	6 months	Low
Amiri M[56]	HOMA-IR	Women with PCOS	COC (EE 30 mcg + DSG 150 mcg) /Non-users	2	NA	NA	12 months	Low
Amiri M[56]	HOMA-IR	Women with PCOS	COC (EE 35 mcg + DRSP 3 mg) /Non-users	5	NA	NA	6 months	Low
Amiri M[56]	HOMA-IR	Women with PCOS	COC (EE 35 mcg + DRSP 3 mg) /Non-users	2	NA	NA	12 months	Low
Amiri M[56]	Fasting insulin	Women with PCOS	COC (EE 35 mcg + CPA 2 mg) /Non-users	4	NA	NA	3 months	Low
Amiri M[56]	Fasting insulin	Women with PCOS	COC (EE 35 mcg + CPA 2 mg) /Non-users	4	NA	NA	6 months	Low
Amiri M[56]	Fasting insulin	Women with PCOS	COC (EE 35 mcg + CPA 2 mg) /Non-users	2	NA	NA	12 months	Low
Amiri M[56]	HOMA-IR	Women with PCOS	COC (EE 35 mcg + CPA 2 mg) /Non-users	3	NA	NA	6 months	Low
Amiri M[56]	HOMA-IR	Women with PCOS	COC (EE 35 mcg + CPA 2 mg) /Non-users	2	NA	NA	12 months	Low
Ralph L[62]	HIV risk	Sub Saharan African women aged 16-50 years	COC/Non-HC user	4	NA/3262	NA/9737	1-2 years	Low

© 2022 Brabaharan S et al. *JAMA Network Open*.

Ralph L[62]	HIV risk	Sub Saharan African women aged 16-50 years	NET-EN (injection)/Non-HC user	2	86/1390	225/5457	1 year	Low
Morrison CS[63]	HIV risk	Sub Saharan African women aged 16-50 years	DMPA/COC user	3	NA/1462	NA/2023	2-2.8 years	Low
Amiri M[56]	SBP	Women at reproductive age with PCOS	COC (EE 35 mcg + DRSP 3 mg) /Non-users	2	NA	NA	6 months	Low
Amiri M[56]	DBP	Women at reproductive age with PCOS	COC (EE 35 mcg + DRSP 3 mg) /Non-users	2	NA	NA	6 months	Low
Amiri M[56]	SBP	Women at reproductive age with PCOS	COC (EE 35 mcg + DRSP 3 mg) /Non-users	2	NA	NA	12 months	Low
Amiri M[56]	DBP	Women at reproductive age with PCOS	COC (EE 35 mcg + DRSP 3 mg) /Non-users	2	NA	NA	12 months	Low
Draper[58]	SBP	Women at reproductive age	DMPA/NET-EN	2	NA/871	NA/492	1-2 years	High
Draper[58]	DBP	Women at reproductive age	DMPA/NET-EN	2	NA/871	NA/492	1-2 years	High
Amiri M[56]	LDL-C	Women with PCOS	COC (EE 30 mcg+ DSG 150 mcg)/Non-users	2	NA	NA	6 months	Low
Amiri M[56]	TG	Women with PCOS	COC (EE 35 mcg + CPA 2 mg)/Non-users	3	NA	NA	3 months	Low
Amiri M[56]	TG	Women with PCOS	COC (EE 35 mcg + CPA 2 mg)/Non-users	2	NA	NA	6 months	Low
Amiri M[56]	TC	Women with PCOS	COC (EE 35 mcg + CPA 2 mg)/Non-users	4	NA	NA	3 months	Low
Amiri M[56]	LDL-C	Women with PCOS	COC (EE 35 mcg + CPA 2 mg)/Non-users	4	NA	NA	3 months	Low
Amiri M[56]	LDL-C	Women with PCOS	COC (EE 35 mcg + CPA 2 mg)/Non-users	3	NA	NA	6 months	Low

© 2022 Brabaharan S et al. *JAMA Network Open*.

Amiri M[56]	HDL-C	Women with PCOS	COC (EE 30 mcg + CPA 2 mg)/Non-users	4	NA	NA	3 months	Low
Amiri M[56]	LDL-C	Women with PCOS	COC (EE 30 mcg + DRSP 3 mg)/Non-users	3	NA	NA	6 months	Low
Amiri M[56]	HDL-C	Women with PCOS	COC (EE 30 mcg + DRSP 3 mg)/Non-users	2	NA	NA	12 months	Low

© 2022 Brabaharan S et al. *JAMA Network Open*.

eTable 5. Nonsignificant Associations From Meta-analyses of RCTs

Source	Health outcome	Population	Intervention (I)	Comparison (C)	Follow up (range)	No. of studies	Total no of participants	Metric	ES (95% CI)	P Value	GRADE evidence (Not serious (NS), serious (S), very serious (VS)						AMSTAR 2
											ROB	I2/ inconstancy	Indirectness	Imprecision	Egger's p-value/ publication bias	Overall certainty of evidence	
Amiri M 2017[56]	BMI change	Women of reproductive age with PCOS	EE 35 mcg + CPA 2 mg	Non-users	3 months	5	81	MD	0.17 (-0.47 to 0.82)[UA]	0.60	S	NS	NS	NS	0.24/No	Moderate	Low
Amiri M 2017[56]	BMI change	Women of reproductive age with PCOS	EE 35 mcg + CPA 2 mg	Non-users	6 months	5	132	MD	-0.28 (-1.13 to 0.57)[UA]	0.52	S	VS	NS	S	0.43/No	Very low	Low
Amiri M 2017[56]	BMI change	Women of reproductive age with PCOS	EE 35 mcg + CPA 2 mg	Non-users	12 months	3	75	MD	-0.63 (-1.60 to 0.34)[UA]	0.20	S	NS	NS	S	NA	Low	Low
Amiri M 2017[56]	BMI change	Women of reproductive age with PCOS	EE 35 mcg + DRSP 3 mg	Non-users	6 months	3	163	MD	-0.18 (-0.87 to 0.51)[UA]	0.61	S	VS	NS	S	0.61/No	Very low	Low
Amiri M 2017[56]	BMI change	Women of reproductive age with PCOS	EE 35 mcg + DRSP 3 mg	Non-users	12 months	3	136	MD	-1.94 (-4.23 to 0.36)[UA]	0.10	S	VS	NS	S	0.92/No	Very low	Low
Lopez LM 2008[61]	Weight increase	Women at reproductive age>18 years	Vaginal ring etonogestrel 120 mcg/EE 15 mcg	COC (LNG 150 mcg/EE 30 mcg)	11-13 months	2	1132	RR	1.70 (0.19-15.48)[NR]	0.64	NS	S	S	VS	NA	Very low	High
Draper 2006[58]	Weight gain	Women at reproductive age	DMPA	NET-EN	12 months	2	1363	MD	0.37 (-0.33 to 1.07)[NR]	0.30	NS	NS	NS	S	NA	Moderate	High
Gallo MF 2013[64]	Depression	Women aged 18-39 years	EE 20mcg + GSD 75 mcg	EE 30 mcg + GSD 75 mcg	12-13 months	2	717	OR	2.12 (0.80-5.66)[UA]	0.13	S	NS	NS	S	NA	Low	High

© 2022 Brabaharan S et al. *JAMA Network Open*.

French R 2001[60]	Ectopic pregnancy	Women aged 18-40 years	LNG-IUS 20 (Mirena ®)	Nova-T IUD ®	1 year	2	3079	RR	0.70 (0.04-11.12)[NR]	0.80	S	NS	NS	S	NA	Low	High
French R 2001[60]	Ectopic pregnancy	Mixed parity	Progestasert ®	Non hormonal IUD	1 year	2	708	RR	3.85 (0.43-34.7)[NR]	0.23	S	NS	S	VS	NA	Very low	High
Chin J 2009[55]	Endometrial fibroids	Post/pre+post-menopausal women with adjuvant tamoxifen for breast cancer	LNG-IUS + Endometrial surveillance	Endometrial surveillance	1-2 years	3	314	OR	0.48 (0.16-1.48)[NR]	0.20	NS	NS	S	S	0.83/No	Moderate	High
Lopez LM 2014[61]	FBG at cycle 6	Reproductive aged women without diabetes	EE 30/DSG	EE 30/GSD	6 months	2	57	MD	0.34 (-3.99 to 4.67)[NR]	0.88	NS	NS	S	S	NA	Low	Critically low
Lopez LM 2014[61]	FBG at cycle 6	Reproductive aged women without diabetes	EE 30/DSG	EE 30/LNG	6 months	2	72	MD	0.20 (0-0.41)[NR]	0.05	VS	NS	S	NS	NA	Very low	Critically low
Lopez LM 2014[61]	FBG at cycle 6	Reproductive aged women without diabetes	EE 35/Norethindrone	EE 30-40/LNG	6 months	2	144	MD	-0.88 (-2.83 to 1.06)[NR]	0.37	S	NS	S	NS	NA	Low	Critically low
Lopez LM 2014[61]	FBG at cycle 12	Reproductive aged women without diabetes	EE 30/DSG	EE 30/LNG	12 months	2	62	MD	0.15 (-0.08 to 0.38)[NR]	0.20	VS	NS	S	NS	NA	Very low	Critically low
Lopez LM 2014[61]	Glucose AUC at cycle 6	Reproductive aged women without diabetes	EE 35/Norethindrone	EE 30-40/LNG	6 months	2	144	MD	-10.83 (-29.62 to 7.96)[NR]	0.26	S	NS	S	VS	NA	Very low	Critically low

© 2022 Brabaharan S et al. *JAMA Network Open*.

Amiri M 2017[56]	FBG 6 months	Women with PCOS	OC users	Non-users	3-24 months	2	88	MD	-0.93 (-3.73 to 1.86)	0.51	S	S	NS	S	NA	NA	Low
Amiri M 2017[56]	FBG 12 months	Women with PCOS	COC (EE 30 mcg + DSG 150 mcg)	Non-users	12 months	2	76	MD	-1.23 (-9.46 to 11.92)	0.82	S	VS	NS	VS	NA	Very low	Low
Amiri M 2017[56]	Fasting insulin 6 months	Women with PCOS	COC (EE 30 mcg + DSG 150 mcg)	Non-users	6 months	2	88	MD	0.80 (-1.42 to 3.03)	0.48	S	NS	NS	S	NA	Low	Low
Amiri M 2017[56]	HOMA-IR	Women with PCOS	COC (EE 30 mcg + DSG 150 mcg)	Non-users	6 months	2	88	MD	0.08 (-0.41 to 0.57)	0.74	S	NS	NS	S	NA	Low	Low
Amiri M 2017[56]	HOMA-IR	Women with PCOS	COC (EE 30 mcg + DSG 150 mcg)	Non-users	12 months	2	76	MD	0.40 (-0.67 to 1.46)	0.47	S	VS	NS	S	NA	Very low	Low
Amiri M 2017[56]	HOMA-IR	Women with PCOS	COC (EE 35 mcg + DRSP 3 mg)	Non-users	6 months	5	193	MD	-0.23 (-0.57 to 0.11)	0.19	S	S	NS	S	0.63/No	Very low	Low
Amiri M 2017[56]	HOMA-IR	Women with PCOS	COC (EE 35 mcg + DRSP 3 mg)	Non-users	12 months	2	116	MD	-0.09 (-0.83 to 0.65)	0.82	S	S	NS	S	NA	Very low	Low
Amiri M 2017[56]	Fasting insulin	Women with PCOS	COC (EE 35 mcg + CPA 2 mg)	Non-users	3 months	4	81	MD	-1.29 (-3.31 to 0.73)	0.21	S	S	NS	S	0.88/No	Very low	Low
Amiri M 2017[56]	Fasting insulin	Women with PCOS	COC (EE 35 mcg + CPA 2 mg)	Non-users	6 months	4	106	MD	1.35 (-0.38 to 3.08)	0.13	NS	NS	NS	S	0.46/No	Moderate	Low
Amiri M 2017[56]	Fasting insulin	Women with PCOS	COC (EE 35 mcg + CPA 2 mg)	Non-users	12 months	2	74	MD	3.00 (-3.19 to 9.19)	0.34	S	VS	NS	S	NA	Very low	Low
Amiri M 2017[56]	HOMA-IR	Women with PCOS	COC (EE 35 mcg + CPA 2 mg)	Non-users	6 months	3	97	MD	0.41 (-0.31 to 1.13)	0.27	NS	NS	NS	S	0.95/No	Moderate	Low

© 2022 Brabaharan S et al. *JAMA Network Open*.

Amiri M 2017[56]	HOMA-IR	Women with PCOS	COC (EE 35 mcg + CPA 2 mg)	Non-users	12 months	2	74	MD	0.70 (-0.30 to 1.70)	0.17	S	VS	NS	S	NA	Very low	Low
Amiri M 2017[56]	LDL-C	Women with PCOS	COC (EE 30 mcg+ DSG 150 mcg)	Non-users	6 months	2	44	MD	14.68 (-1.25 to 30.61)	0.07	VS	VS	NS	VS	NA	Very low	Low
Amiri M 2017[56]	TG	Women with PCOS	COC (EE 35 mcg + CPA 2 mg)	Non-users	3 months	3	66	MD	-4.13 (-13.00 to 4.75)	0.36	VS	NS	NS	S	0.05/Yes	Very low	Low
Amiri M 2017[56]	TG	Women with PCOS	COC (EE 35 mcg + CPA 2 mg)	Non-users	6 months	2	55	MD	9.93 (-0.62 to 20.48)	0.07	VS	NS	NS	VS	NA	Very low	Low
Amiri M 2017[56]	TC	Women with PCOS	COC (EE 35 mcg + CPA 2 mg)	Non-users	3 months	4	76	MD	10.82 (-15.80 to 37.44)	0.43	VS	VS	NS	VS	0.34/No	Very low	Low
Amiri M 2017[56]	LDL-C	Women with PCOS	COC (EE 35 mcg + CPA 2 mg)	Non-users	3 months	4	76	MD	3.62 (-13.4 to 20.66)	0.68	VS	VS	NS	VS	0.13/No	Very low	Low
Amiri M 2017[56]	LDL-C	Women with PCOS	COC (EE 35 mcg + CPA 2 mg)	Non-users	6 months	3	65	MD	3.41 (-22.80 to 29.50)	0.8-	VS	VS	NS	VS	0.88/No	Very low	Low
Amiri M 2017[56]	HDL-C	Women with PCOS	COC (EE 30 mcg + CPA 2 mg)	Non-users	3 months	4	76	MD	4.30 (-2.75 to 11.36)	0.23	VS	VS	NS	S	0.01/Yes	Very low	Low
Amiri M 2017[56]	LDL-C	Women with PCOS	COC (EE 30 mcg + DRSP 3 mg)	Non-users	6 months	3	121	MD	0.82 (-7.47 to 9.11)	0.85	VS	S	NS	S	0.63/No	Very low	Low
Amiri M 2017[56]	HDL-C	Women with PCOS	COC (EE 30 mcg + DRSP 3 mg)	Non-users	12 months	2	79	MD	6.23 (-1.11 to 13.58)	0.10	VS	VS	NS	S	NA	Very low	Low
Ralph L 2015[62]	HIV risk	Sub Saharan African women aged 16-50 years	COC	Non-HC user	1-2 years	4	12999	HR	0.90 (0.73-1.11)	0.32	S	NS	NS	NS	0.55/No	Moderate	Low
Ralph L 2015[62]	HIV risk	Sub Saharan African women aged 16-50 years	NET-EN (injection)	Non-HC user	1 year	2	6847	HR	1.19 (0.88-1.61)	0.27	S	NS	NS	S	NA	Low	Low

© 2022 Brabaharan S et al. *JAMA Network Open*.

Morrison CS 2015[63]	HIV risk	Sub Saharan African women aged 16-50 years	DMPA	COC user	2-2.8 years	3	3485	HR	1.23 (0.87-1.74)	0.23	S	NS	NS	S	0.62/No	Low	Low
Amiri M 2007[56]	SBP	Women at reproductive age with PCOS	COC (EE 30 mcg + DRSP 3 mg)	Non-users	6 months	2	116	MD	0.69 (-3.79 to 5.16)[UA]	0.76	S	S	NS	S	NA	Very low	Low
Amiri M 2007[56]	DBP	Women at reproductive age with PCOS	COC (EE 30 mcg + DRSP 3 mg)	Non-users	6 months	2	116	MD	-1.93 (-9.34 to 5.47)[UA]	0.61	S	VS	NS	S	NA	Very low	Low
Amiri M 2007[56]	SBP	Women at reproductive age with PCOS	COC (EE 30 mcg + DRSP 3 mg)	Non-users	12 months	2	116	MD	-0.87 (-4.99 to 3.25)[UA]	0.68	S	NS	NS	S	NA	Low	Low
Amiri M 2007[56]	DBP	Women at reproductive age with PCOS	COC (EE 30 mcg + DRSP 3 mg)	Non-users	12 months	2	116	MD	-2.10 (-11.48 to 7.29)[UA]	0.66	S	VS	NS	VS	NA	Very low	Low
Draper H 2006[58]	SBP	Women at reproductive age	DMPA	NET-EN	1-2 years	2	1363	MD	-2.31 (-8.79 to 4.16)[NR]	0.48	NS	VS	NS	S	NA	Very low	High
Draper H 2006[58]	DBP	Women at reproductive age	DMPA	NET-EN	1-2 years	2	1363	MD	-0.57 (-1.81 to 0.66)[NR]	0.36	NS	NS	NS	S	NA	Moderate	High

Abbreviations: [NR] = adjusted or unadjusted effect size is not reported; [UA] = unadjusted effect size; AUC= Area under the curve; BMI = Body Mass Index; CI = Confidence interval; CPA = Cyproterone acetate; COC = Combined oral contraceptive; DBP = Diastolic blood pressure; DMPA = Depot medroxyprogesterone acetate; DRSP = Drospirenone; DSG = Desogestrel; EE = Ethniyl estradiol; ES = Effect size; FBG = Fasting blood glucose; HIV = Human immunodeficiency virus; HDL = High-density lipoprotein; HOMA-IR = Homeostatic Model Assessment for Insulin Resistance; HC = Hormonal contraceptive; HR = Hazard ratio; IUS =Intrauterine system; LDL-C = Low-density lipoprotein; LNG-IUS = Levonorgestrel-intrauterine system; MD = Mean difference; NA = Not available; NET-EN; Norethisterone enanthate; NS= Not serious; OR = odds ratio; PCOS = Polycystic ovarian syndrome; RR = Risk ratio; TC = Total cholesterol; S = Serious; SBP = Systolic blood pressure; VS = Very serious

© 2022 Brabaharan S et al. *JAMA Network Open*.

eTable 6. Sensitivity Analyses of Meta-analysis of RCTs Initially Graded as High or Moderate

Outcome	Population	Primary analysis			Sensitivity analysis: Excluding high ROB trials			Sensitivity analysis: excluding small size studies (25th percentile)			Sensitivity analysis: HKSJ method (<5 studies)		
		No of studies	ES (95% CI)	GRADE	No of studies	ES (95% CI)	GRADE	No of studies	ES (95% CI)	GRADE	No of studies	ES (95% CI)	GRADE
[a]Endometrial polyps	Post/Pre- + Post-menopausal women with adjuvant tamoxifen for breast cancer	4	OR: 0.22 (0.13-0.38)[NR]	High	NA	NA	NA	NA	NA	NA	4	0.22 (0.09-0.54)[NR]	High
[b]***Sub group***: Endometrial polyps	Post -menopausal women with adjuvant tamoxifen for breast cancer	2	OR: 0.30 (0.14-0.65)[NR]	High	NA	NA	NA	NA	NA	NA	Not applicable for subgroup analysis		
[c]FBG at 6-month	Women of reproductive age with PCOS	5	MD: -2.05 (-2.82 to -1.28)	Moderate	3	[d]MD: -3.03 (-4.92 to -1.14)	High	NA	NA	NA	NA	NA	NA

a: RoB, Inconsistency, Imprecision & Indirectness: Not serious. Publication bias – – no (Egger's test: 0.731)
b: RoB, Inconsistency, Imprecision & Indirectness: Not serious. Publication bias – undetected.
c: RoB: Serious; Inconsistency & Indirectness: Not serious; Imprecision: Not serious: Publication bias – – no (Egger's test: 0.221)
d: RoB, Inconsistency, Imprecision & Indirectness: Not serious. Publication bias – – no (Egger's test: 0.948)

Note: [NR] = adjusted or unadjusted effect size is not reported; CI: Confidence interval; ES: Effect size; FBG; Fasting blood glucose; GRADE: Grading of Recommendation, Assessment, Development and Evaluations; HKSJ: Hartung-Knapp-Sidik-Jonkman; MD: Mean difference; NA: Not applicable; OR: Odds Ratio; PCOS: Polycystic ovarian syndrome; ROB: Risk of bias

© 2022 Brabaharan S et al. *JAMA Network Open*.

eTable 7. Descriptive Characteristics of Included Meta-analyses of Cohort Studies

Author; publication year	Outcomes	No of studies	Total number of participants	Adjustment for confounding variables
Significant associations				
Moorman PG 2013[65]	Breast cancer	2	NA	Age, parity, family cluster, history of oophorectomy before right censoring, age at menarche, breastfeeding, year of birth
Moorman PG 2013[65]	Breast cancer	2	NA	Age, parity, family cluster, history of oophorectomy before right censoring, age at menarche, breastfeeding, year of birth
Moorman PG 2013[65]	Breast cancer	2	NA	Age, age at first pregnancy, marital status, parity or number of pregnancies, age at menarche, breastfeeding, year of birth
Amiri M 2017[56]	BMI	3	228	Not adjusted
Asthana 2020[66]	Cervical cancer	5	NA	Age, parity, BMI, marital status, level education, physical activity, smoking habit, OC use and duration, menopausal status with hormonal replacement therapy (HRT) use, social class, smoking, influence of age at first marriage, age at first-term pregnancy, ever diaphragm and condom use
Asthana 2020[66]	Cervical cancer	5	NA	Age, parity, BMI, marital status, level education, physical activity, smoking habit, OC use and duration, menopausal status with HRT use, social class, smoking, influence of age at first marriage, age at first-term pregnancy, ever diaphragm and condom use
Asthana 2020[66]	Cervical cancer	4	NA	Age, parity, BMI, marital status, level education, physical activity, smoking habit, OC use and duration, menopausal status with HRT use, social class, smoking
Delgado-Rodriguez 1992[67]	Carcinoma in situ	5	NA	Age, race, age at first intercourse, parity or number of pregnancies, socioeconomic status, marital status, age at first pregnancy, number of previous pap smears, history of sexually transmitted disease and tobacco use
Luan NN 2015[68]	Colorectal cancer adenoma	8	NA	Age, smoking status, diabetes, BMI, physical activity, alcohol use, menopausal status, pack years of smoking, education, parity, social class
Bosetti C 2009[69]	Colorectal cancer adenoma	6	NA	Age, parity, smoking, social class, duration use of HRT, BMI, menopausal status, pack years of smoking, education, randomized treatment assignment, family history of colorectal cancer, previous history of benign colorectal polyps, physical activity, aspirin use, alcohol consumption, intake of red meat, multivitamin use, baseline postmenopausal hormone use, age at menarche, total energy intake, height, parity, total vitamin E intake, a total vitamin E by age interaction term and vitamin A supplement intake, randomized treatment assignment, family history of colorectal cancer, previous history of benign colorectal polyps
Bosetti C 2009[69]	Colorectal cancer adenoma	6	NA	Age, parity, smoking, social class, duration use of HRT, BMI, menopausal status, pack years of smoking, education, randomized treatment assignment, family history of colorectal cancer, previous history of benign colorectal polyps, physical activity, aspirin use, alcohol consumption, intake of red meat, multivitamin use, baseline postmenopausal hormone use, age at menarche

© 2022 Brabaharan S et al. *JAMA Network Open*.

Xu J-L 2015[70]	Dry socket	16	2470	Not adjusted
Vercellini 2011[71]	Endometriosis	5	NA	Age, parity, adjusted for age (months), calendar time (2-year questionnaire period), and body mass index at age 18 years, race, family history of endometriosis, time to regular menstrual periods and length of menstrual cycle.
Vercellini 2011[71]	Endometriosis	5	NA	Age, parity, adjusted for age (months), calendar time (2-year questionnaire period), and body mass index at age 18 years, race, family history of endometriosis, time to regular menstrual periods and length of menstrual cycle
Shere M 2015[72]	Plasma folate concentration	12	2038	Not reported
Shere M 2015[72]	RBC folate concentration	9	956	Not reported
Lan Y 2018[(68)]	Glioma risk	4	NA	Age, study center, randomization group, parity, age at menarche (<12, 13–14, >14), menopausal status, smoking status, alcohol intake, socioeconomic level, parity, age at first birth, OC use, education, BMI
Liu H 2017[74]	HTN (SBP ≥ 140mm Hg and DBP ≥ 90mm Hg)	3	58324	Age, BMI, smoking status, regular care, parity, alcohol intake, ethnicity, family history of hypertension, and physical activity
Ortizo R 2017[75]	IBD	5	68382	Age, social class, smoking, sex, calendar year, OA, RA, depression, anxiety, stress, asthma, chronic obstructive pulmonary disease, diabetes, irritable bowel syndrome and appendectomy
Ortizo R 2017[75]	Crohn's Disease	5	68382	Age, social class, smoking, sex, calendar year, OA, RA, depression, anxiety, stress, asthma, chronic obstructive pulmonary disease, diabetes, irritable bowel syndrome and appendectomy
Wang X 2019[48]	Ulcerative colitis	3	295484	Age, social class, smoking, cohort, BMI, parity, age at menarche, HRT use and menopause status
Liu H 2014[76]	Kidney cancer	4	NA	Age, age at first life birth, age at menarche, pack-years, BMI, menopausal status, education, study center, randomization group, parity, HRT use, smoking status, hypertension, alcohol intake, diuretic use, fruit intake, vegetable intake and race
Amiri M 2017[56]	Triglycerides	3	107	Age and BMI
Amiri M 2017[56]	Triglycerides	2	45	Age and BMI
Amiri M 2017[56]	Total cholesterol	4	118	Age and BMI
Halperin J 2011[77]	Triglycerides	6	119	Quality of studies, age, BMI and duration of study
Havrilesky 2013[78]	Ovarian cancer	7	NA	Age, parity, menopausal status, HT, country, BMI, smoking, social class, height, age at first term pregnancy, age at first marriage, family history, breastfeeding, education, physical activity, other contraception methods, unilateral oophorectomy and hysterectomy
Havrilesky 2013[78]	Ovarian cancer	5	NA	Age, parity, menopausal status, HT, country, BMI, smoking, social class, height, age at first term pregnancy, age at first marriage, unilateral oophorectomy and hysterectomy
Xu 2015 [79]	Ischemic stroke	3	1083196	Smoking, hypertension, diabetes, obesity, alcohol intake, lipid level

© 2022 Brabaharan S et al. *JAMA Network Open*.

Johnston 1998[80]	Mortality due to subarachnoid hemorrhage (SAH)	2	51144	Race, smoking, socioeconomic status
Pérez-López FR 2020[81]	Suicide risk	3	167689	Calendar time, age, BMI, smoking status, race, weight change, height, alcohol intake, physical activity, diet, SBP, use of antihypertensives drugs, history of diabetes, parenteral myocardial infarction, before age of 60 and time since menopause, parity, social class
Baratloo A 2014[82]	VTE	3	NA	Current OCP user, calendar year, educational level, age, BMI, smoking, cancer, fractures, surgery, use of warfarin
Bateson D 2016[83]	VTE	6	NA	Age, site, year of entry into study, diabetes, hypertension, hyperlipidemia, smoking, obesity, history of cancer, calendar year, length of schooling and education, duration of oral contraceptive use, smoking, ovarian stimulation drugs, recent surgery, EE dose, user type (current/new), BMI, insurance status, history of hormonal contraceptive use, history of use of other sexual hormones/modulators, history of pregnancy, childbirth & puerperium period, heart diseases, follow up examination after surgery
Martinez F 2012[84]	VTE	4	283719	Age, calendar year, length of schooling and education, duration of oral contraceptive use, smoking, ovarian stimulation drugs, recent surgery, BMI, insurance status, history of hormonal contraceptive use, history of use of other sexual hormones/modulators, history of pregnancy, childbirth & puerperium period, heart diseases, follow up examination after surgery
Dragoman MV 2018[85]	VTE	2	NA	Age, year and level of education, BMI and thrombophilia,
Mantha S 2012[86]	VTE	3	NA	Age, duration of use, calendar year, length of schooling and education, length of oral contraceptive use, smoking, ovarian stimulation drugs, recent surgery
Oedingen C 2018[87]	VTE	3	NA	Smoking, BMI, age, duration of use, calendar year and education
Martinez F 2012[84]	VTE	8	503821	Age, calendar year, length of schooling and education, length of oral contraceptive use, smoking, ovarian stimulation drugs, recent surgery
Martinez F 2012[84]	VTE	5	340729	Age, calendar year, length of schooling and education, duration of oral contraceptive use, smoking, ovarian stimulation drugs, recent surgery, BMI, insurance status, history of hormonal contraceptive use, history of use of other sexual hormones/modulators, history of pregnancy, childbirth & puerperium period, heart diseases, follow up examination after surgery
Oedingen C 2018[87]	VTE	3	256815	Age, calendar year, length of schooling and education, length of oral contraceptive use, smoking, ovarian stimulation drugs, recent surgery
Non-significant associations				
Zhu H 2012[88]	Breast cancer	13	NA	Age, smoking, alcohol drinking, educational level, BMI, age at menarche, parity and family history of breast cancer, number of children, age at 1st pregnancy, duration of breastfeeding, smoking, exercise, obesity, energy consumption, geographical area, invitation to do breast screening, menopausal status, HRT use, BMI, alcohol consumption, age at first birth, history of benign breast disease, marital status, maternal history of breast cancer
Nindrea 2019[89]	Breast cancer	2	7440	Age at recruitment, marital status, family history of cancer, breastfeeding history, genetic risk score, age at first live birth, age at menarche, family history, past breast biopsy, BMI

© 2022 Brabaharan S et al. *JAMA Network Open*.

Conz 2020[90]	Breast cancer	2	NA	Age, education, endometriosis, parity, family history of premenopausal breast or ovarian cancer, age at the start of follow-up, female hormones in the form of oral contraceptives (OC), fertility drugs, or HT or prophylactic use of tamoxifen
Goshtasebi A[91]	Peak spinal BMD accrual 12 month	2	346	Age, race, body weight, tobacco use, physical activity, baseline BMD, BMI, weight-bearing physical activity, calcium intake and self-reported menstruation regularity when not using OC/pregnant/nursing
Goshtasebi A[91]	Peak spinal BMD accrual 24 month	3	462	Age, race, height, body weight, tobacco use, 24-hour physical activity (kcal/d) baseline BMD, BMI, BMI change over 2 hours, weight-bearing physical activity, calcium intake, age at menarche, alcohol consumption, total calcium intake and self-reported menstruation regularity when not using OC/pregnant/nursing
Amiri M 2017[56]	BMI	4	199	NA
Amiri M 2017[56]	BMI	4	151	NA
Asthana 2020[66]	Cervical cancer	4	91	Age, height, age at first-term pregnancy, social class, smoking, parity, BMI, influence of age at first marriage, ever use of HRT, ever diaphragm use, ever condom use
Delgado-Rodriguez 1992[67]	Dysplasia	4	248	Age, race, age at first intercourse, parity or number of pregnancies, socioeconomic status, age at first pregnancy,
Delgado-Rodriguez 1992[67]	Invasive cervical cancer	3	NA	Age, age at first pregnancy, marital status, parity or number of pregnancies
Song J 2019[92]	Colorectal adenoma	4	742097	Age, race, education, BMI, smoking, alcohol, anti-inflammatory drug use, history of previous colorectal endoscopy and mutually adjusted other reproductive factors, family history of colorectal cancer, aspirin, NSAIDs, physical activity, sitting watching TV/VCR, calcium, beef/pork/lamb as main dish, region, strenuous exercise
Song J 2019[92]	Colorectal adenoma	2	NA	Age, race, education, BMI, smoking, alcohol, anti-inflammatory drug use, mutually adjusted other reproductive factors, age at endoscopy, previous history of endoscopy before 1980, family history of colorectal adenoma, physical activity, aspirin use, intake of alcohol, red meat, folate, methionine and use of postmenopausal hormones
Amiri M 2017[56]	FBG 3 months	3	108	Age and BMI
Amiri M 2017[56]	FBG 6 months	2	151	Age and BMI
Amiri M 2017[56]	FBG 6 months	3	199	Age and BMI
Amiri M 2017[56]	Fasting insulin 3 months	2	158	Age and BMI
Amiri M 2017[56]	Fasting insulin 6 months	2	125	Age and BMI
Amiri M 2017[56]	HOMA-IR 3 months	2	119	Age and BMI
Amiri M 2017[56]	HOMA-IR 6 months	2	116	Age and BMI
Amiri M 2017[56]	HOMA-IR 6 months	2	241	Age and BMI

© 2022 Brabaharan S et al. *JAMA Network Open*.

Morrison CS 2015[63]	HIV infection	3	4513	Age, married/living with partner, number of sex partners, condom use, region, education level, parity, workplace, duration of sex work, vaginal washing practices, number of sexual partners per week, incident genital tract infection
Morrison CS 2015[63]	HIV infection	2	3832	Age, married/living with partner, number of sex partners, condom use, region, education level, parity, workplace, duration of sex work, vaginal washing practices, number of sexual partners per week, incident genital tract infection
Morrison CS 2015[63]	HIV infection	2	3757	Age, married/living with partner, laboratory-confirmed infection, number of sex partners, condom use, region, education level, parity, workplace, duration of sex work, vaginal washing practices, number of sexual partners per week, incident genital tract infection
Amiri M 2017[56]	SBP 6 months	2	57	NA
Amiri M 2017[56]	DBP 6 months	2	57	NA
Liu H 2014[76]	Kidney cancer	5	1581	Age, BMI, waist to hip ratio, alcohol use, history of hypertension, pack-years, BMI, menopausal status, education, study center, randomization group, diuretic use, fruit intake, vegetable intake, race, smoking status
Amiri M 2017[56]	Triglycerides	2	19	Age and BMI
Amiri M 2017[56]	Total cholesterol	2	19	Age and BMI
Amiri M 2017[56]	Total cholesterol	2	45	Age and BMI
Amiri M 2017[56]	LDL-Cholesterol	3	107	Age and BMI
Amiri M 2017[56]	LDL-Cholesterol	2	19	Age and BMI
Amiri M 2017[56]	LDL-Cholesterol	2	45	Age and BMI
Amiri M 2017[56]	HDL-Cholesterol	3	107	Age and BMI
Amiri M 2017[56]	HDL-Cholesterol	2	19	Age and BMI
Amiri M 2017[56]	HDL-Cholesterol	2	45	Age and BMI
Halperin J 2011[77]	HDL-Cholesterol	9	269	Quality of studies, age, BMI, estrogen dose
An N 2015[93]	Liver cancer	3	NA	Age, alcohol, BMI, diabetes, race, smoking, social status, parent cohort study, menopausal status, education, parity, having had a tubal ligation
Stampfer 1990[94]	Non-fatal MI and fatal coronary disease	3	NA	Age, parenteral history of infarction before age of 60, menopausal status, hormone use, time period, smoking status, hypertension, diabetes, high cholesterol, age, Quetelet index,
Peragallo Urrutia 2013[95]	MI	2	NA	Age, parity, BMI, smoking, social class, education, alcohol intake, physical activity, history of hypertension, history of diabetes, menopausal status
Khader YS 2003[96]	MI	2	NA	Social class and smoking
Khader YS 2003[96]	MI	2	NA	Social class and smoking

© 2022 Brabaharan S et al. *JAMA Network Open*.

Lagergreen K 2014[97]	Esophageal adenocarcinoma	2	356116	Age, study type, ethnicity, BMI, reflux, tobacco smoking, alcohol intake, education, BMI, physical activity and fruit vegetable intake, total energy and hysterectomy
Havrilesky 2013[78]	Ovarian cancer	3	NA	Age, parity, menopausal status, HT, country, BMI, smoking, social class, height, age at first term pregnancy, age at first marriage, family history, age at menarche, breastfeeding, education, physical activity, other contraception methods
Qi S 2014[98]	Rheumatoid arthritis	5	332897	Age, parity, smoking, social class, age, follow up cycle, age at menarche, time since menopause, BMI
Chen Q 2014[99]	Rheumatoid arthritis	3	NA	Age, parity, cigarette smoking, weight, social class, follow up cycle, time since menopause, BMI, age at menarche
Chen Q 2014[99]	Rheumatoid arthritis	4	NA	Age, cigarette smoking, weight, parity, social class, follow up cycle, BMI
Pladevall-Villa 1996[100]	Rheumatoid arthritis	2	NA	Age, cigarette smoking, weight, follow up cycle, age at menarche, parity, time since menopause, BMI
Xu 2018[101]	Hemorrhagic stroke	5	602620	Smoking, hypertension, alcohol, socioeconomic status, race
Li F 2019[102]	Stroke	7	1326249	Age, educational level, calendar year, diabetes, hypertension, hyperlipidemia, arrhythmia, smoking, BMI, pack-years of smoking, physical activity, high blood pressure, Quetelet index
Wu L 2015[103]	Thyroid cancer	9	1291941	Age, study center, parity, age at recruitment, duration, ethnicity, age at menarche, BMI, age at menopause, hormone therapy, physical activity, height, observational study/clinical trial (CT), alcohol intake, pack years of smoking, and history of goiter/nodules, randomization status in each CT, education, family history of thyroid cancer, marital status, number of live births, cumulative breast-feeding months and other contraceptive methods, alcohol consumption, time since last pregnancy
Dragoman MV 2018[85]	VTE risk	2	NA	Age, year, level of education, fibroids, endometriosis, menstrual disorders, hypertension, hyperlipidemia, CVD, diabetes, asthma, back pain, recent emergency room visits and recent physician visits
Dayan N 2011[104]	VTE risk	2	235	BMI, age, thrombophilia
De Bastos M 2014[105]	VTE risk	3	NA	Age, year, level of education
Glisic M 2018[106]	VTE risk	3	NA	Age, thrombophilia, BMI, calendar year and education
Oedingen C 2018[87]	VTE risk	3	277414	Age, duration of use, calendar year, length of schooling and education, smoking, ovarian stimulation drugs, recent surgery
Oedingen C 2018[87]	VTE risk	3	926752	Age, calendar year, length of schooling and education, length of oral contraceptive use, smoking, ovarian stimulation drugs, recent surgery, complementary universal health insurance, medical risk factors, gynecological visit during previous year

© 2022 Brabaharan S et al. *JAMA Network Open*.

eTable 8. Nonsignificant Associations From Meta-analyses of Cohort Studies

Source	Adverse health outcome	Population	Exposed	Unexposed	Number of studies per association	Follow-up (range)	Metric	Random effect measure, ES (95% CI)	No of cases	P value	I^2 (%)	Largest study (95% CI)	PrI (95% CI)	SSE	10% CCT (95% CI)	Amstar
Zhu H 2012[84)]	Breast cancer	Pre- and post-menopausal women	Ever OC user	Never user	13	5-28 years	OR	1.08 (0.99-1.17)	11424	0.10	61.4	1.06 (1.00-1.23)	0.82-1.41	0.77	0.96-1.10	Critically low
Nindrea 2019[89]	Breast cancer	Women of reproductive age	Ever OC user (>5 years)	Never user	2	1 cohort:11 years	OR	1.15 (0.61-2.15)	145	0.67	54	1.46 (0.98-2.18)	NA	NA	0.58-2.11	Moderate
Conz 2020[90]	Breast cancer	Women aged <50 years	LNG user	Never user	2	10-17 years	OR	1.15 (0.98-1.34)	7314	0.09	50.6	1.21 (1.11-1.32)	NA	NA	0.91-1.29	High
Goshtasebi A 2019[91]	Peak spinal BMD accrual	Women aged 12-19 years	CHC user	Never user	2	12 months	MD	[a]-0.01 (-0.03 to 0.01)	NA	0.41	67.5	0 (-0.01 to 0.01)	NA	NA	-0.03 to 0.01	Moderate
Goshtasebi A 2019[91]	Peak spinal BMD accrual	Women aged 12-19 years	CHC user	Never user	3	24 months	MD	-0.02 (-0.04 to 0.00)	NA	0.06	90.2	-0.04 (-0.05 to -0.03)	-0.61 to 0.57	0.74	-0.04 to 0.001	Moderate
Amiri M 2017[56]	BMI changes	Women of reproductive age	EE + CPA	Never user	4	3 months	MD	0.61 (-0.96 to 2.17)[UA]	NA	0.45	0	0.09 (-1.05 to 1.23)	-40.53 to 15.75	0.42	-0.74 to 0.91	Critcally low
Amiri M 2017[56]	BMI changes	Women of reproductive age	EA + CPA	Never user	4	6 months	MD	0.01 (-2.19 to 2.21)[UA]	NA	0.99	0	0.10 (-2.50 to 2.70)	NA	NA	-2.19 to 2.21	Critically low
Delgado-Rodriguez 1992[67]	Dysplasia	Women	Ever OC user	Non hormonal contracepti	4	1-42 years	OR	1.88 (0.98-3.60)	91	0.06	51	2.30 (0.80-7.10)	0.20-17.93	0.01	0.86-2.43	Critcally low

© 2022 Brabaharan S et al. *JAMA Network Open*.

				ve users												
Delgado-Rodriguez[67]	Carcinoma in situ	Women	Ever OC user	Non hormonal contraceptive users	4	3-12 years	OR	1.20 (0.94-1.55)	248	0.15	8.6	1.44 (1.03-2.02)	0.65-2.20	0.61	0.85-1.49	Critically low
Delgado-Rodriguez[67]	Invasive cervical cancer	Women	Ever OC user	Non hormonal contraceptive users	3	6-20 years	OR	4.09 (0.88-19.03)	>20	0.07	45.4	1.80 (1.00-3.30)	NA	0.07	0.92-5.10	Critically low
Song J 2019[92]	CRC adenoma	Women aged 30-74 years	Ever OC user	Never user	4	14-24 years	OR	0.99 (0.89-1.10)	>3000	0.79	72.3	0.93 (0.86-1.01)	0.62-1.56	0.74	0.89-1.07	High
Song J 2019[92]	CRC adenoma	Women aged 30-55 years	Ever OC user (> 5 years)	Never user	2	14-22 years	OR	1.09 (0.97-1.22)	1341	0.15	0	1.10 (0.98-1.25)	NA	NA	0.95-1.24	High
Amiri M 2017[56]	FBG	Reproductive age women with PCOS	EE + CPA	Never user	3	3 months	WMD	1.44 (-1.40 to 4.28)	NA	0.32	66	3.60 (1.47-3.53)	-29.78 to 32.66	0.77	-1.58 to 2.33	Critcally low
Amiri M 2017[56]	FBG	Reproductive age women with PCOS	EE + CPA	Never user	2	6 months	WMD	0.34 (-5.40 to 6.09)	NA	0.91	0	0 (-7.83 to 7.83)	NA	NA	-5.40 to 6.09	Critically low
Amiri M 2017[56]	FBG	Reproductive age women with PCOS	EE + DRSP	Never user	3	6 months	WMD	0.28 (-5.91 to 6.47)	NA	0.93	69.7	-4.50 (-9.10 to 0.10)	-171 to 171.56	0.54	-5.28 to 5.78	Critically low
Amiri M 2017[56]	Fasting insulin	Reproductive age women with PCOS	EE + CPA	Never user	2	3 months	WMD	0.39 (-1.81 to 2.58)	NA	0.73	0	0.55 (-2.06 to 3.16)	NA	NA	-1.81 to 2.58	Critcally low

© 2022 Brabaharan S et al. *JAMA Network Open*.

Amiri M 2017[56]	Fasting insulin	Reproductive age women with PCOS	EE + CPA	Never user	2	6 months	WMD	0.75 (-3.44 to 1.95)	NA	0.59	0	0.61 (-3.94 to 2.72)	NA	NA	-3.44 to 1.948	Critically low
Amiri M 2017[56]	HOMA-IR 3 months	Reproductive age women with PCOS	EE + CPA	Never user	2	3 months	WMD	0.09 (-0.44 to 0.62)	NA	0.74	0	0.11 (-0.49 to 0.71)	NA	NA	-0.44 to 0.62	Critically low
Amiri M 2017[56]	HOMA-IR 6 months	Reproductive age women with PCOS	EE + CPA	Never user	2	6 months	WMD	-0.15 (-0.74 to 0.43)	NA	0.60	0	-0.10 (-0.83 to 0.63)	NA	NA	-0.744 to 0.43	Critically low
Amiri M 2017[56]	HOMA-IR 6 months	Reproductive age women with PCOS	EE + DRSP	Never user	2	6 months	WMD	0.14 (-0.88 to 1.16)	NA	0.78	93.6	0.64 (0.43-0.85)	NA	NA	-0.96 to 1.06	Critically low
Morrison CS 2015[63]	HIV infection	Sub-Saharan African women aged 15-49 yeas	DMPA user	Non-hormonal contraceptive user	3	1-2 years	HR	1.44 (0.83-2.52)	355	0.20	70	1.22 (0.86-1.72)	NA	0.63	0.78-1.98	Moderate
Morrison CS 2015[63]	HIV infection	Sub-Saharan African women aged 15-49 years	COC user	Non-hormonal contraceptive user	2	1-2 years	HR	1.19 (0.82-1.72)	285	0.35	39.6	1.01 (0.70-1.45)	NA	NA	0.82-1.73	Moderate
Morrison CS 2015[63]	HIV infection	Sub-Saharan African women aged 15-49 years	DMPA user	COC user	2	1-2 years	HR	1.27 (0.97-1.66)	275	0.09	0	1.21 (0.87-1.67)	NA	NA	0.96-1.67	Moderate

© 2022 Brabaharan S et al. *JAMA Network Open*.

Amiri M 2017[56]	SBP 6 months	Women of reproductive age with PCOS	CPA 2 mg/EE 35 mcg	Non-OC user	2	6 months	MD	1.67 (-3.06 to 6.39)[UA]	NA	0.49	0	2.00 (-3.01 to 7.01)	NA	NA	-3.06 to 6.40	Critically low
Amiri M 2017[56]	DBP 6 months	Women of reproductive age with PCOS	CPA 2 mg/EE 35 mcg	Non-OC user	2	6 months	MD	0.89 (-2.88 to 4.66)[UA]	NA	0.64	0	1.00 (-3.00 to 5.00)	NA	NA	-2.88 to 4.66	Low
Liu H 2014[74]	Kidney cancer	Women of reproductive age	Ever OC user	Never user	5	12-28 years	SRR	0.90 (0.81-1.01)	1581	0.08	0	0.87 (0.75-1.02)	0.75-1.09	0.38	0.80-1.05	Moderate
Amiri M 2017[56]	TG	Reproductive age women with PCOS	EE + CPA	Never user	2	6 months	WMD	17.92 (-8.32 to 44.15)	NA	0.18	32.4	26.57 (5.59-47.55)	NA	NA	-16.82 to 41.87	Critically low
Amiri M 2017[56]	TC	Reproductive age women with PCOS	EE + CPA	Never user	2	6 months	WMD	11.75 (-22.45 to 45.96)	NA	0.50	74.2	27.07 (9.62-44.52)	NA	NA	-24.47 to 40.56	Critically low
Amiri M 2017[56]	TC	Reproductive age women with PCOS	EE + DRSP	Never user	2	6 months	WMD	31.19 (-18.11 to 80.49)	NA	0.22	90.8	55.40 (39.25-71.55)	NA	NA	-23.21 to 49.81	Critically low
Amiri M 2017[56]	LDL	Reproductive age women with PCOS	EE + CPA	Never user	3	3 months	WMD	4.80 (-0.59 to 10.18)	NA	0.08	0	2.05 (-5.64 to 9.74)	-135.50 to 145.09	0.50	-2.12 to 9.57	Critically low
Amiri M 2017[56]	LDL	Reproductive age women with PCOS	EE + CPA	Never user	2	6 months	WMD	10.25 (-2.73 to 23.23)	NA	0.12	0	11.61 (-3.40 to 26.62)	NA	NA	-2.73 to 23.23	Critically low

© 2022 Brabaharan S et al. *JAMA Network Open*.

Amiri M 2017[56]	LDL	Reproductive age women with PCOS	EE + DRSP	Never user	2	6 months	WMD	13.75 (-21.93 to 49.43)	NA	0.45	87.8	30.70 (19.31-42.09)	NA	NA	-27.36 to 40.06	Critically low
Amiri M 2017[56]	HDL	Reproductive age women with PCOS	EE + CPA	Never user	3	3 months	WMD	6.26 (-7.21 to 19.73)	NA	0.36	96.5	10.31 (6.83-13.79)	-374.80 to 387.30	0.41	-12.45 to 20.58	Critically low
Amiri M 2017[56]	HDL	Reproductive age women with PCOS	EE + CPA	Never user	2	6 months	WMD	9.74 (-3.03 to 22.50)	NA	0.14	79.3	15.47 (10.32-20.62)	NA	NA	-5.12 to 14.13	Critically low
Amiri M 2017[56]	HDL	Reproductive age women with PCOS	EE + DRSP	Never user	2	6 months	WMD	11.61 (-3.87 to 27.09)	NA	0.14	85.5	3.80 (-4.20 to 11.80)	NA	NA	-2.88 to 12.58	Critically low
Halperin J 2011[77]	HDL	Heathy women	OC user	Never user	9	3-12 months	WMD	0.36 (-0.13 to 0.85)	NA	0.15	99.3	1.21 (1.41-1.28)	-2.58 to 3.30	0.36	-0.05 to 0.28	Critically low
An N 2015[93]	Liver cancer	Women aged <30->70 years	OC user	Never OC user	3	11-36 years	RR	0.88 (0.64-1.22)	696	0.45	46.9	0.82 (0.60-1.13)	0.04-22.18	0.50	0.67-1.23	Critically low
Stampfer 1990[94]	Non-fatal MI and fatal coronary disease	Female aged 25-79 years	Past CHC user	Non-users	3	8 years	RR	0.85 (0.69-1.05)	209	0.13	0	0.80 (0.60-1.00)	0.21-3.35	0.80	0.67-1.12	Critically low
Peragallo Urrutia 2013[95]	MI	Female of reproductive age	Current OC user	Non-concurrent user	2	11-18 years	RR	0.97 (0.46-2.02)	106	0.93	41.4	0.70 (0.35-1.40)	NA	NA	0.46-2.02	Critically low
Khader YS 2003[96]	MI	Female aged 15-55 years	Current OC user	Never user	2	1 study reported: 6.5 years	RR	1.45 (0.71-2.93)	NA	0.93	15.9	1.80 (0.90-3.60)	NA	NA	0.65-2.91	Critically low

© 2022 Brabaharan S et al. *JAMA Network Open*.

Khader YS 2003[96]	MI	Female aged 15-55 years	Past OC user	Never user	2	1 study reported: 6.5 years	RR	0.95 (0.66-1.36)	NA	0.76	0	1.00 (0.70-1.60)	NA	NA	0.66-1.36	Critically low
Lagergreen K 2014[97]	Oesophageal adenocarcinoma	Women aged 50-79 years	OC user	Non-OC user	2	1-5 years	OR	0.84 (0.55-1.31)	88	0.45	0	0.88 (0.53-1.46)	NA	NA	0.55-1.31	Low
Havrilesky 2013[78]	Ovarian cancer	Women aged 25-71 years	Ever OC user (<1 year use)	Non-users	3	2-7.5 years	OR	1.24 (0.90-1.70)	477	0.19	14	1.36 (0.87-2.13)	0.11-14.29	0.93	0.88-1.66	Moderate
Qi S 2014[98]	RA	Women aged 16-59 years	OC user (past + current)	Never user	5	1-26 years	RR	1.03 (0.91-1.60)	1285	0.69	0	1.10 (0.90-1.30)	0.84-1.25	0.84	0.91-1.16	Low
Chen Q 2014[99]	RA	Women aged 16-55 years	OC current user	Never user	3	1-20 years	RR	0.96 (0.69-1.34)	295	0.83	12.2	0.82 (0.59-1.15)	0.07-12.91	0.54	0.69-1.34	Critically low
Chen Q 2014[99]	RA	Women aged 16-55 years	OC past user	Never user	4	1-20 years	RR	0.93 (0.78-1.12)	635	0.45	0	0.94 (0.72-1.22)	0.63-1.38	0.73	0.78-1.12	Critically low
Pladevall-Vila 1996[100]	RA	Women aged 25-55 years	OC user (longest duration)	Never user	2	8-15 years	RR	1.19 (0.65-2.16)	140	0.57	66.2	0.90 (0.60-1.40)	NA	NA	0.63-1.98	Critically low
Xu 2018[101]	Hemorrhagic stroke	Women aged 21-70 years	Current OCP user	Non-current user	5	2.9-12.9 years	OR	1.10 (0.42-2.88)	1730	0.84	85.4	0.36 (0.18-0.70)	0.03-37.56	0.69	0.560-2.08	Critically low
Li 2019[102]	Stroke	Women aged 15-19 years	OCP duration (every 5-year increment)	Non-user	7	8.8-18.6 years	OR	1.19 (0.92-1.54)	5529	0.19	88.5	1.99 (1.71-2.31)	0.49-2.49	0.29	0.96-1.18	Low
Wu L 2015[103]	Thyroid cancer	Women of reproductive age	Longest duration of OC	Shortest duration of OC	9	7.5-15.9 years	RR	0.85 (0.71-1.02)	1479	0.08	26.1	0.66 (0.50-0.89)	0.58-1.25	0.61	0.79-1.11	Moderate

© 2022 Brabaharan S et al. *JAMA Network Open*.

Dragoman MV 2018[85]	VTE	Women of reproductive age	NRG user	LNG user	2	1 cohort:7 years	OR	1.50 (0.93-1.43)	370	0.19	0	1.20 (0.90-1.60)	NA	NA	0.32-1.43	High
Dayan N 2011[104]	VTE	Women aged 13-44 years	OCP users with thrombophilia	Non-users without thrombophilia	2	1 cohort:33 months	OR	11.05 (0.47-258.48)	39	0.15	74.4	51.33 (6.79-388.35)	NA	NA	0.34-30.79	Low
De Bastos M 2014[101])	VTE	Women aged 12-50 years	1st gen COC user	Non-User	3	5-36 years	OR	1.45 (0.70-2.98)	>1890	0.32	61.1	0.94 (0.44-1.98)	NA	0.36	0.70-1.78	High
Glisic M 2017[106]	VTE	Women aged 15-53 years	Oral POC	Non-user	3	5-13 years	OR	0.81 (0.45-1.48)	1863	0.50	3.8	1.30 (0.50-3.00)	0.01-49.7	0.98	0.46-1.59	High
Oedingen C 2018[87]	VTE	Women aged 15-49 years	DSG-COC/20 E2	LNG-COC	3	2-36 years	OR	1.06 (0.77-1.45)	2096	0.74	64	1.04 (0.86-1.25)	0.44-30.40	0.38	0.83-1.19	Critically low
Oedingen C 2018[87]	VTE	Women aged 15-49 years	GSD-COC/20EE	LNG-C0C	3	2-8 years	OR	1.07 (0.89-1.29)	1805	0.47	49.9	1.05 (0.86-1.29)	0.16-7.29	0.96	0.88-1.21	Critically low

Abbreviations: [NR] = adjusted or unadjusted effect size is not reported; [UA] = unadjusted effect size; BMD = Bone mineral density; BMI = Body mass index; CCT = Credibility ceiling test; CE = Class of evidence; CHC = Combined hormonal contraceptive; CI = Confidence interval; COC = Combined oral contraceptive; CPA = Cyproterone acetate; DMPA = Depot medroxyprogesterone acetate; DRSP = Drospirenone; DSG = Desogestrel; EE = Ethniyl estradiol; ES = Effect size; ESB = Excess significance bias; FBG = Fasting blood glucose; GSD = Gestodene; HC = Hormonal contraceptive; HDL = High-density lipoprotein; HIV = Human immunodeficiency virus; HOMA-IR = Homeostatic Model Assessment for Insulin Resistance; HR = Hazard ratio; HTN = Hypertension; IBD = Inflammatory bowel disease; IUS =Intrauterine system; LDL-C = Low-density lipoprotein; LNG = Levonorgestrel; LNG-IUS = Levonorgestrel-intrauterine system; MD = Mean difference; MI = Myocardial infarction; NA = Not applicable; NP = Not pertinent; NRG = Norgestimate; OC = Oral contraceptive; OR = Odds ratio; PCOS = Polycystic ovarian syndrome; POC = Progesterone-only contraceptive; PrI = Prediction interval; RA = Rheumatoid arthritis; RR = Risk ratio; SAH = Subarachnoid hemorrhage; SRR = Summary risk ratio; SSE = Small study effect; TC = Total cholesterol; TG= Triglycerides; VTE = Venous thromboembolism; WMD = Weighted mean difference.

© 2022 Brabaharan S et al. *JAMA Network Open*.

NA = not applicable because of non-significant effect estimate or the data is not available; NP = not pertinent, because estimated number is larger than observed, and there is no evidence of excess significance based on assumption made for plausible effect size

© 2022 Brabaharan S et al. *JAMA Network Open*.

eTable 9. Sensitivity and Subgroup Analyses of Meta-analysis of Cohort Studies Initially Graded as Convincing or Highly Suggestive

Outcome	Population	Primary analysis			Sensitivity analysis: Excluding low quality trials			Sensitivity analysis: excluding small size studies (25th percentile)			Sensitivity analysis: HKSJ method (<5 studies)		
		No of studies	ES (95% CI)	CE	No of studies	ES (95% CI)	CE	No of studies	ES (95% CI)	CE	No of studies	ES (95% CI)	CE
[a]Endometriosis	Asymptomatic women/Women who undergone surgery for endometriosis	5	RR:1.60 (1.40-1.82)	Class I	NA	NA	NA	NA	NA	NA	NA	NA	NA
[b]***Sub group***: Endometriosis	Asymptomatic women	3	RR: 1.52 (1.09-2.12)	Class IV	NA	NA	NA	NA	NA	NA	NA	NA	NA
[c]VTE risk	Women of reproductive age	6	RR: 2.05 (1.59-2.64)	Class II	2	[d]RR: 2.52 (1.89-3.37)	Class IV	NA	NA	NA	NA	NA	NA
[e]VTE risk	Women of reproductive age	3	RR: 2.42 (1.76-3.32)	Class II	3	RR: 2.42 (1.76-3.32)	Class II	NA	NA	NA	3	RR:2.42 (1.26-4.68)	Class IV

a: Inconsistency: <50%; No of cases: >1000; Largest study effect: Significant; PrI: Significant; P value: 1.32×10^{-12}; ESB: Not significant; 10% CCT: Significant; Publication bias – – no (Egger's test: 0.338)
b: Inconsistency: <50%; No of cases: 213; Largest study effect: Not significant; PrI: Not significant; P value: 4.94×10^{-4}; ESB: NP; 10% CCT: Not significant; Publication bias – – no (Egger's test: 0.997)
c: Inconsistency: >50%; No of cases: >1000; Largest study effect: Significant; PrI: Not significant; P value: 2.66×10^{-8}; ESB: NA; 10% CCT: Significant; Publication bias – – yes (Egger's test: 0.019)
d: Inconsistency: >50%; No of cases: >1000; Largest study effect: Significant; PrI: NA; P value: 3.10×10^{-10}; ESB: NA; 10% CCT: Not significant; Publication bias – – undetected
e: Inconsistency: >50%; No of cases: >1000; Largest study effect: Significant; PrI: Not significant; P value: 4.54×10^{-8}; ESB: NA; 10% CCT: Significant; Publication bias – – no (Egger' test: 0.504)

Note: CCT: Credibility ceiling test; CE: Class of evidence; CI: Confidence interval; ES: Effect size; ESB: Excess significance bias; HKSJ: Hartung-Knapp-Sidik-Jonkman; NA: Not applicable; NP: Not pertinent; PrI: Prediction interval; RR: Risk ratio; VTE: Venous thromboembolism

© 2022 Brabaharan S et al. *JAMA Network Open*.

eFigure 1. PRISMA Flow Diagram

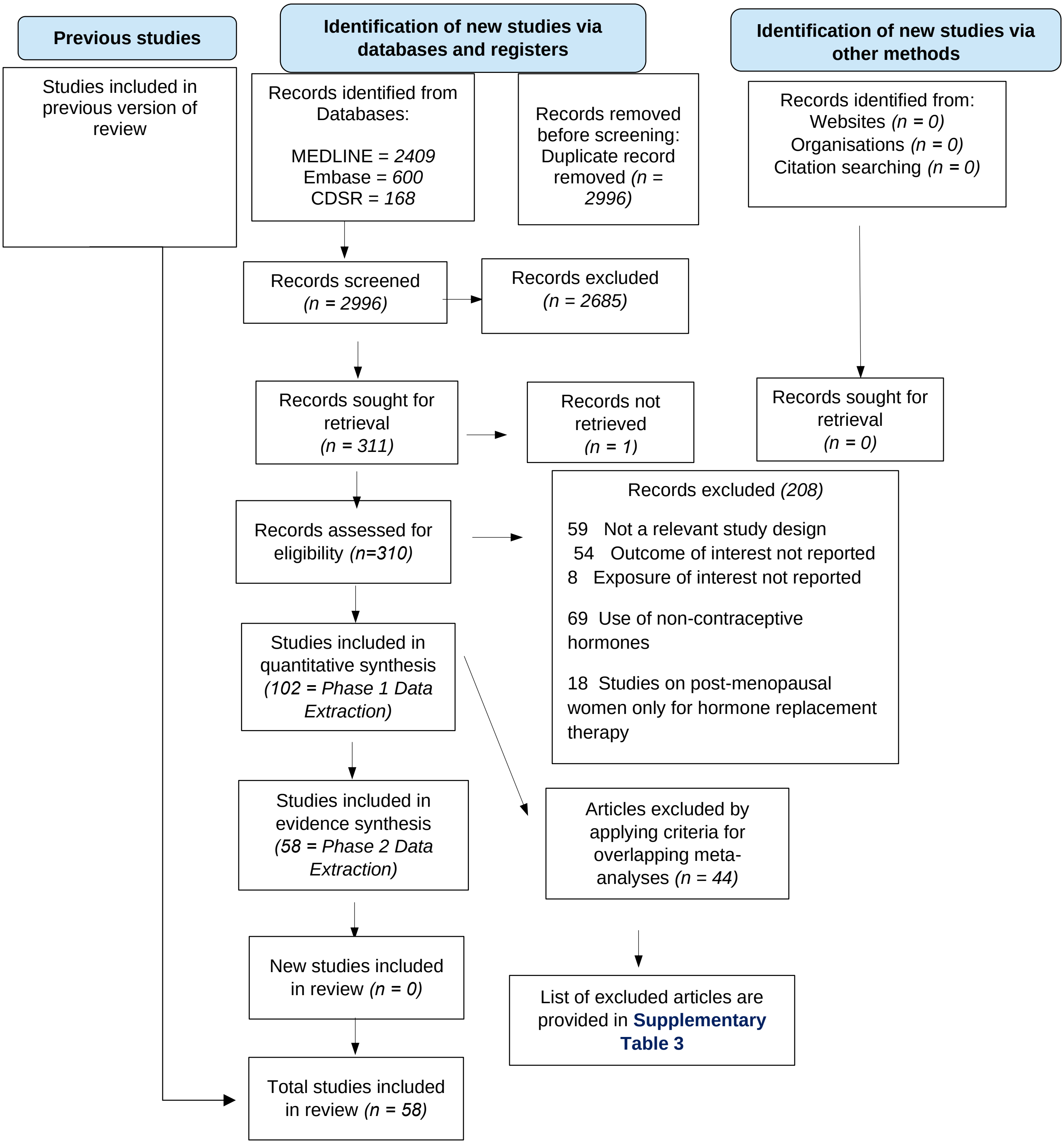

© 2022 Brabaharan S et al. *JAMA Network Open*.

eFigure 2. Forest Plot of Association Between LNG-IUS and Risk of Endometrial Polyps

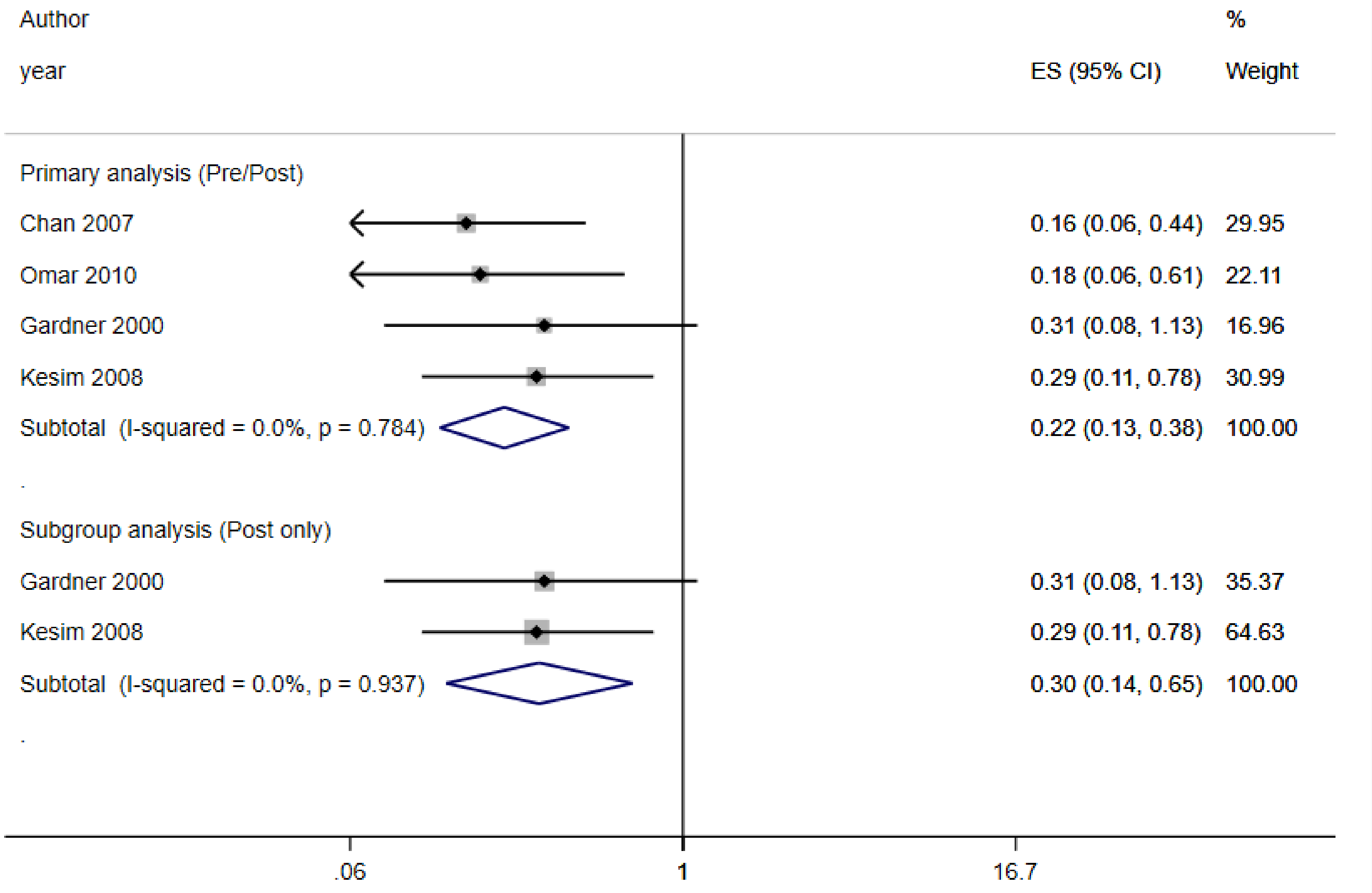

Abbreviations: CI = Confidence interval; ES = Effect size; LNG-IUS = Levonorgestrel-intrauterine system

Predictive interval for primary analysis: 0.07-0.73; for subgroup analysis: no predictive interval is generated due to <3 studies.

Note: The effect sizes reported are not stated as adjusted or unadjusted in their original meta-analyses' individual studies.

© 2022 Brabaharan S et al. *JAMA Network Open*.

eFigure 3. Forest Plot of Association Between COC (EE, 30 mcg/CPA) and FBG Levels

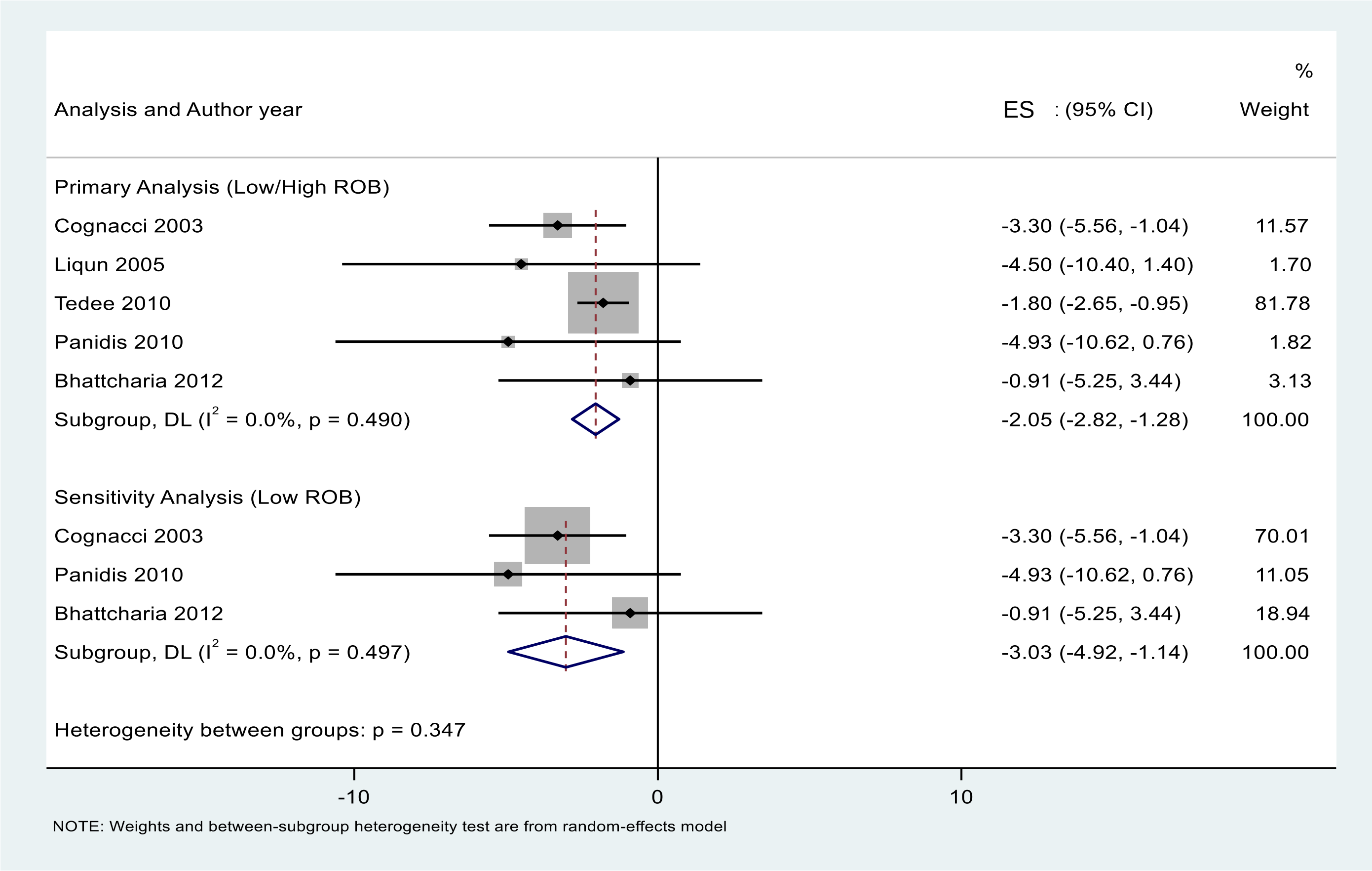

Abbreviations: CI = Confidence interval; CPA = Cyproterone acetate; COC = Combined oral contraceptive; ES = Effect size; FBG; Fasting blood glucose; ROB = Risk of bias

Predictive interval for primary analysis: -3.32 to -0.84

Predictive interval for sensitivity analysis: -15.29 to 9.23

Note: The effect sizes reported are not stated as adjusted or unadjusted in their original meta-analyses' individual studies.

© 2022 Brabaharan S et al. *JAMA Network Open*.

eFigure 4. Forest Plot of Association Between Past Oral Contraceptive Users and Endometriosis Risk

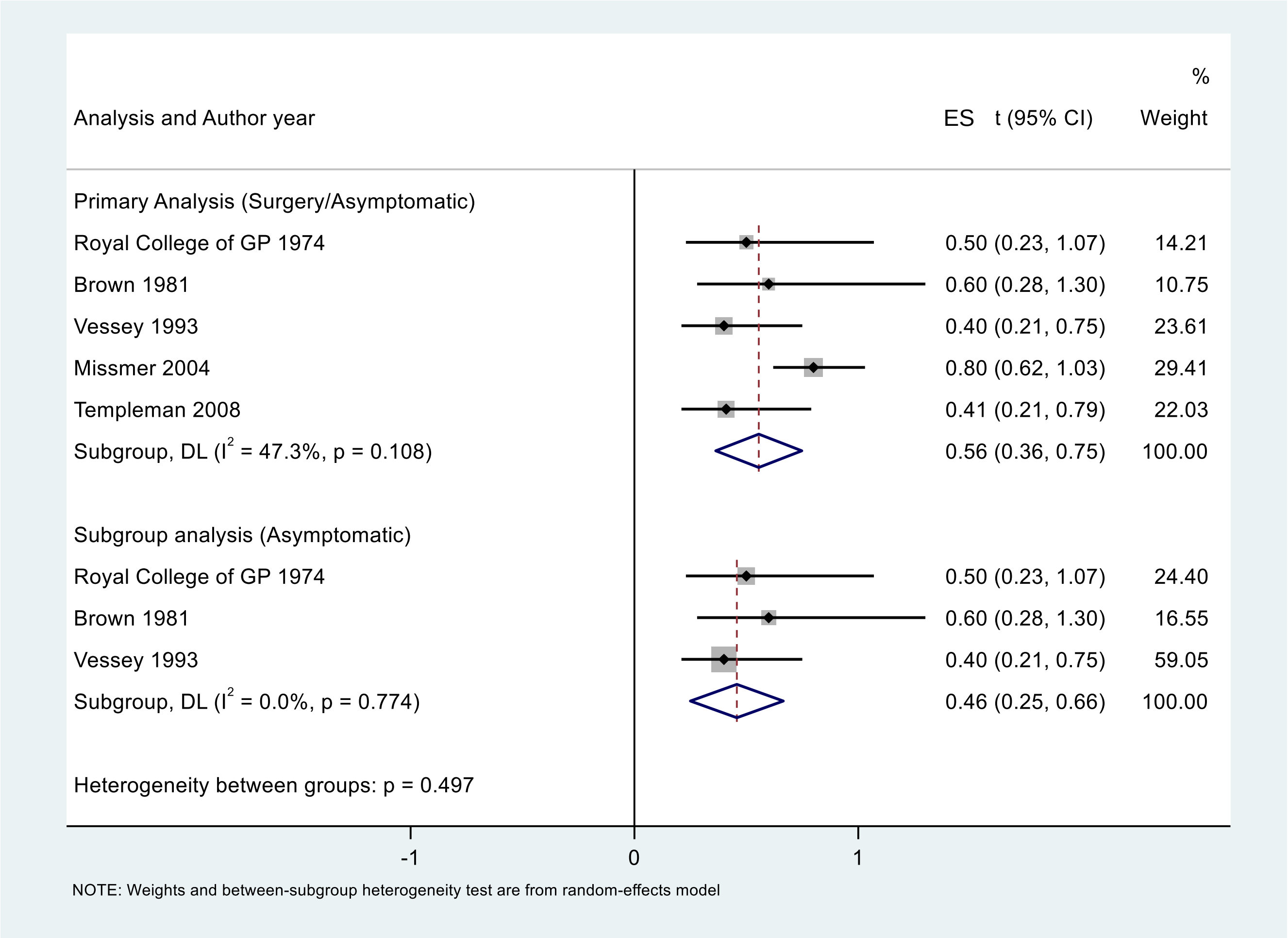

Abbreviations: CI = Confidence interval; ES = Effect size; OC = Oral contraceptive

Predictive interval for primary analysis: 0.21-1.51

Predictive interval for subgroup analysis: 0.03 -6.99

© 2022 Brabaharan S et al. *JAMA Network Open*.

eFigure 5. Forest Plot of Association Between DSG-COC Low Dose and VTE Risk

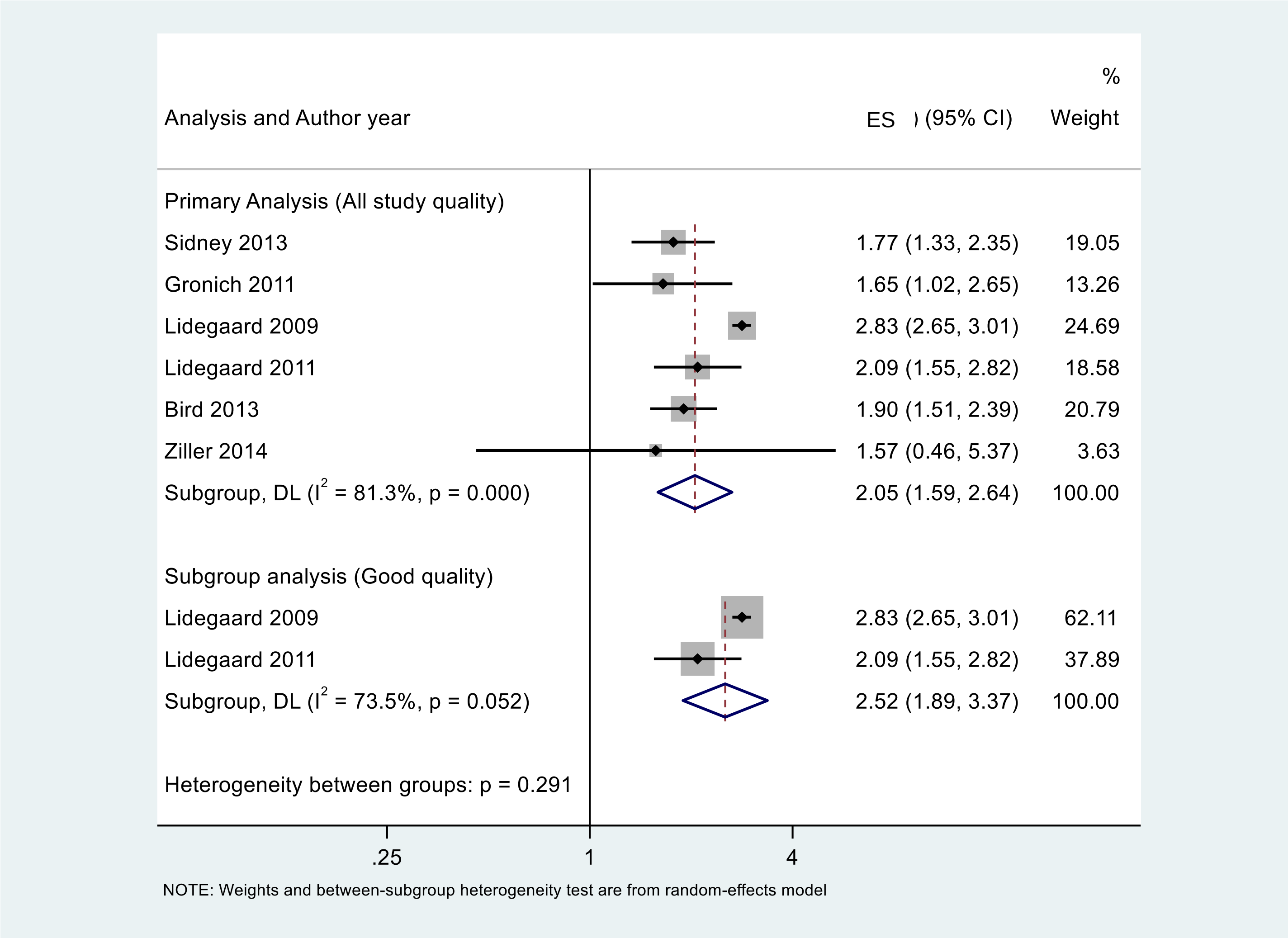

Abbreviations: CI = Confidence interval; COC = Combined oral contraceptive; DSG = Desogestrel; ES = Effect size; VTE = Venous thromboembolism

Predictive interval for primary analysis: 0.92-4.57; for subgroup analysis: no predictive interval is generated due to <3 studies.

© 2022 Brabaharan S et al. *JAMA Network Open*.

eFigure 6. Schematic Network Diagram of Associations in RCTs (Binary Data)

Clinical Outcome	Intervention	Indication	Comparison	No of Studies	Metric	ES	LCI	UCI	I^2 (%)	AMSTAR-2	GRADE
**Increased risk**											
Weight gain[66]	LNG-IUS	HMB	Ablation	2	RR	2.60[NR]	1.16	5.84	0	High	Moderate
HIV risk[62]	DMPA	Contraception	Non-HC user	4	HR	1.30	1.10	1.53	0	Low	Low
Ovarian cysts[54]	LNG-IUS	Contraception	Other medical treatment	3	RR	3.05[NR]	1.21	7.70	0	High	Very low
**Reduced risk**											
Endometrial polyps[55]	LNG-IUS	Contraception	Non-users	4	OR	0.22[NR]	0.13	0.38	0	High	High

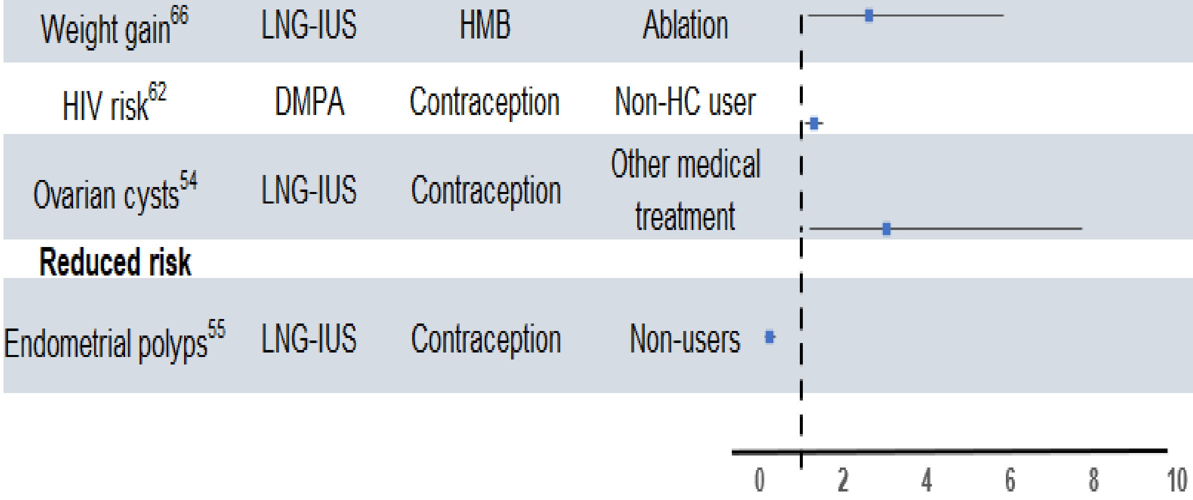

Abbreviations: [NR] = adjusted or unadjusted effect size is not reported; AMSTAR-2 = A Measurement Tool to Assess Systematic Review version 2; CI = Confidence interval; COC = Combined oral contraceptive; DMPA = Depot medroxyprogesterone acetate; ES = Effect size; GRADE = Grading of Recommendation, Assessment, Development and Evaluations; HC = Hormonal contraceptive; HIV = Human immunodeficiency virus; HMB = Heavy menstrual bleed; I^2 = heterogeneity; LCI = Lower confidence interval; LNG-IUS: Levonorgestrel-intrauterine system; UCI = Upper confidence interval

© 2022 Brabaharan S et al. *JAMA Network Open*.

eFigure 7. Schematic Network Diagram of Associations Between COC Users vs Non-COC Users With PCOS in RCTs (Continuous Data)

Clinical Outcome	*Intervention*	*No of Studies*	*Metric*	*ES*	*LCI*	*UCI*	*I^2 (%)*	*AMSTAR-2*	*GRADE*
**Increased risk**									
Fasting insulin[56]	COC (EE 30 mcg + DSG 150 mcg)	2	MD	2.32	1.15	3.49	3	High	Moderate
LDL[56]	COC (EE 35 mcg + CPA 2 mg)	2	MD	15.08	12.74	17.43	0	Low	Low
LDL[56]	COC (EE 30 mcg + DRSP 3 mg)	2	MD	11.53	4.73	18.34	0	Low	Very low
TC[56]	COC (EE 35 mcg + CPA 2 mg)	2	MD	42.20	17.01	67.38	74.4	Low	Very low
**Reduced risk**									
FBG[56]	COC (EE 35 mcg + CPA 2 mg)	5	MD	-2.05	-2.82	-1.28	0	Moderate	Low
FBG[56]	COC (EE 30 mcg + DRSP 3 mg)	4	MD	-4.34	-7.55	-0.93	73.5	Very low	Low
HDL[56]	COC (EE 35 mcg + CPA 2 mg)	2	MD	10.00	8.41	11.59	0	Low	Low
HDL[56]	COC (EE 30 mcg + DRSP 3 mg)	2	MD	6.50	1.91	11.09	47.4	Very low	Low
HOMA-IR[56]	COC (EE 35 mcg + CPA 2 mg)	2	MD	-0.75	-1.24	-0.25	0	Very low	Low
TC[56]	COC (EE 35 mcg + CPA 2 mg)	2	MD	-3.67	-7.26	0.07	0	Very low	Low

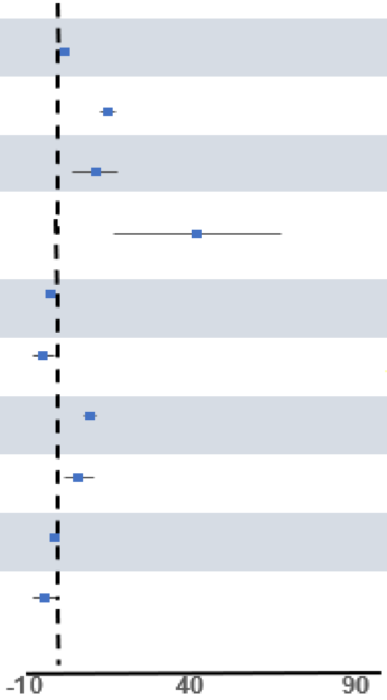

Abbreviations: AMSTAR-2 = A Measurement Tool to Assess Systematic Review version 2; CI = Confidence interval; COC = Combined oral contraceptive; CPA = Cyproterone acetate; DRSP = Drospirenone; DSG = Desogestrel; EE = Ethniyl estradiol; ES = Effect size; FBG = Fasting blood glucose; GRADE = Grading of Recommendation, Assessment, Development and Evaluations; HDL = High-density lipoprotein; HOMA-IR = Homeostatic Model Assessment for Insulin Resistance; I^2 = heterogeneity; LCI = Lower confidence interval; LDL-C = Low-density lipoprotein; LNG-IUS = Levonorgestrel-intrauterine system; MD = Mean difference; PCOS = Polycystic ovarian syndrome; TC = Total cholesterol; UCI = Upper confidence interval

© 2022 Brabaharan S et al. *JAMA Network Open*.

eFigure 8. Schematic Network Diagram of Associations in Cohort Studies

Clinical Outcome	Exposed	Unexposed	No of Studies	Metric	ES	LCI	UCI	I^2 (%)	AMSTAR-2	Class Of Evidence
**Increased risk**										
Endometriosis[71]	OC past user	Never user	5	RR	1.60	1.4	1.82	1.6	Moderate	I
VTE[82]	OC user	Never user	3	OR	2.42	1.76	3.32	73.8	Moderate	II
VTE[83]	DSG-COC (low dose EE)	LNG-user	6	RR	2.05	1.59	2.64	81.3	Moderate	II
VTE[84]	DSG-COC	LNG-COC	8	OR	1.93	1.31	2.85	89.1	Moderate	III
VTE[87]	GSD-COC/30-40 EE	LNG-COC	3	OR	1.45	1.16	1.18	46.9	Critically low	III
**Reduced risk**										
Glioma risk[73]	OC user	Never user	4	OR	0.75[NR]	0.67	0.85	0	Moderate	III

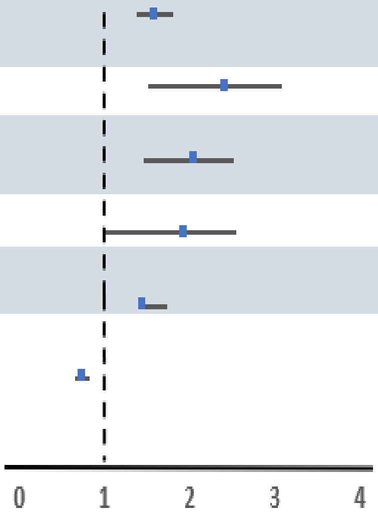

Abbreviations: [NR] = adjusted or unadjusted effect size is not reported; AMSTAR-2 = A Measurement Tool to Assess Systematic Review version 2; CI = Confidence interval; COC = Combined oral contraceptive; DRSP = Drospirenone; DSG = Desogestrel; EE = Ethniyl estradiol; ES = Effect size; GSD = Gestodene; LCI = Lower confidence interval; LNG = Levonorgestrel; OC = Oral contraceptive; OR = Odds ratio; RR = Risk ratio; UCI = Upper confidence interval; VTE = Venous thromboembolism

© 2022 Brabaharan S et al. *JAMA Network Open*.

eReferences

1. Dragioti E, Solmi M, Favaro A, Fusar-Poli P, Dazzan P, Thompson T, et al. Association of Antidepressant Use With Adverse Health Outcomes: A Systematic Umbrella Review. JAMA Psychiatry. 2019 Dec 1;76(12):1241–55.

2. Neuenschwander M, Ballon A, Weber KS, Norat T, Aune D, Schwingshackl L, et al. Role of diet in type 2 diabetes incidence: umbrella review of meta-analyses of prospective observational studies. BMJ. 2019 Jul 3;366:l2368.

3. He Y, Li X, Gasevic D, Brunt E, McLachlan F, Millenson M, et al. Statins and Multiple Noncardiovascular Outcomes: Umbrella Review of Meta-analyses of Observational Studies and Randomized Controlled Trials. Ann Intern Med. 2018 Oct 16;169(8):543–53.

4. Hulot J-S, Collet J-P, Silvain J, Pena A, Bellemain-Appaix A, Barthélémy O, et al. Cardiovascular Risk in Clopidogrel-Treated Patients According to Cytochrome P450 2C19*2 Loss-of-Function Allele or Proton Pump Inhibitor Coadministration: A Systematic Meta-Analysis. J Am Coll Cardiol. 2010 Jul 6;56(2):134–43.

5. Kwok CS, Loke YK. Meta-analysis: the effects of proton pump inhibitors on cardiovascular events and mortality in patients receiving clopidogrel. Aliment Pharmacol Ther. 2010;31(8):810–23.

6. Lin L, Cui B, Deng Y, Jiang X, Liu W, Sun C. The Efficacy of Proton Pump Inhibitor in Cirrhotics with Variceal Bleeding: A Systemic Review and Meta-Analysis. Digestion. 2021;102(2):117–27.

7. Ottawa Hospital Research Institute [Internet]. [cited 2021 Jan 13]. Available from: http://www.ohri.ca/programs/clinical_epidemiology/oxford.asp

8. Shea BJ, Reeves BC, Wells G, Thuku M, Hamel C, Moran J, et al. AMSTAR 2: a critical appraisal tool for systematic reviews that include randomised or non-randomised studies of healthcare interventions, or both. BMJ. 2017 Sep 21;358:j4008.

9. Higgins JPT, Altman DG, Gøtzsche PC, Jüni P, Moher D, Oxman AD, et al. The Cochrane Collaboration's tool for assessing risk of bias in randomised trials. BMJ. 2011 Oct 18;343:d5928.

10. Lopez-Picado A, Lapuente O, Lete I. Efficacy and side-effects profile of the ethinylestradiol and etonogestrel contraceptive vaginal ring: a systematic review and meta-analysis. Eur J Contracept Reprod Health Care Off J Eur Soc Contracept. 2017;22(2):131–46.

11. Cortessis VK, Barrett M, Brown Wade N, Enebish T, Perrigo JL, Tobin J, et al. Intrauterine Device Use and Cervical Cancer Risk: A Systematic Review and Meta-analysis. Obstet Gynecol. 2017;130(6):1226–36.

12. Peng Y, Wang X, Feng H, Yan G. Is oral contraceptive use associated with an increased risk of cervical cancer? An evidence-based meta-analysis. J Obstet Gynaecol Res. 2017;43(5):913–22.

13. Wang S, Wang Y, Xu J, Chen Y. Is the oral contraceptive or hormone replacement therapy a risk factor for cholelithiasis: A systematic review and meta-analysis. Medicine (Baltimore). 2017;96(14):e6556.

© 2022 Brabaharan S et al. *JAMA Network Open*.

14. La Vecchia C, Ron E, Franceschi S, Dal Maso L, Mark SD, Chatenoud L, et al. A pooled analysis of case-control studies of thyroid cancer. III. Oral contraceptives, menopausal replacement therapy and other female hormones. Cancer Causes Control CCC. 1999;10(2):157–66.

15. Hawley W, Nuovo J, DeNeef CP, Carter P. Do oral contraceptive agents affect the risk of breast cancer? A meta-analysis of the case-control reports. J Am Board Fam Pract. 1993;6(2):123–35.

16. Mol BW, Ankum WM, Bossuyt PM, Van der Veen F. Contraception and the risk of ectopic pregnancy: a meta-analysis. Contraception. 1995;52(6):337–41.

17. Romieu I, Berlin JA, Colditz G. Oral contraceptives and breast cancer. Review and meta-analysis. Cancer. 1990;66(11):2253–63.

18. Romieu I, Hernandez-Avila M, Liang MH. Oral contraceptives and the risk of rheumatoid arthritis: a meta-analysis of a conflicting literature. Br J Rheumatol. 1989;28 Suppl 1(b1t, 8302415):13–23.

19. Koster T, Small RA, Rosendaal FR, Helmerhorst FM. Oral contraceptives and venous thromboembolism: a quantitative discussion of the uncertainties. J Intern Med. 1995;238(1):31–7.

20. Spector TD, Hochberg MC. The protective effect of the oral contraceptive pill on rheumatoid arthritis: an overview of the analytic epidemiological studies using meta-analysis. J Clin Epidemiol. 1990;43(11):1221–30.

21. Karagas MR, Stukel TA, Dykes J, Miglionico J, Greene MA, Carey M, et al. A pooled analysis of 10 case-control studies of melanoma and oral contraceptive use. Br J Cancer. 2002;86(7):1085–92.

22. Fernandez E, La Vecchia C, Balducci A, Chatenoud L, Franceschi S, Negri E. Oral contraceptives and colorectal cancer risk: a meta-analysis. Br J Cancer. 2001;84(5):722–7.

23. Hennessy S, Berlin JA, Kinman JL, Margolis DJ, Marcus SM, Strom BL. Risk of venous thromboembolism from oral contraceptives containing gestodene and desogestrel versus levonorgestrel: a meta-analysis and formal sensitivity analysis. Contraception. 2001;64(2):125–33.

24. Rushton L, Jones DR. Oral contraceptive use and breast cancer risk: a meta-analysis of variations with age at diagnosis, parity and total duration of oral contraceptive use. Br J Obstet Gynaecol. 1992;99(3):239–46.

25. Amoozegar F, Ronksley PE, Sauve R, Menon BK. Hormonal contraceptives and cerebral venous thrombosis risk: a systematic review and meta-analysis. Front Neurol. 2015;6(101546899):7.

26. Cibula D, Zikan M, Dusek L, Majek O. Oral contraceptives and risk of ovarian and breast cancers in BRCA mutation carriers: a meta-analysis. Expert Rev Anticancer Ther. 2011;11(8):1197–207.

27. Skegg DC, Noonan EA, Paul C, Spears GF, Meirik O, Thomas DB. Depot medroxyprogesterone acetate and breast cancer. A pooled analysis of the World Health Organization and New Zealand studies. JAMA. 1995;273(10):799–804.

© 2022 Brabaharan S et al. *JAMA Network Open*.

28. Baillargeon J-P, McClish DK, Essah PA, Nestler JE. Association between the current use of low-dose oral contraceptives and cardiovascular arterial disease: a meta-analysis. J Clin Endocrinol Metab. 2005;90(7):3863–70.

29. Gallo MF, Grimes DA, Schulz KF, d'Arcangues C, Lopez LM. Combination injectable contraceptives for contraception. Cochrane Database Syst Rev. 2005;(3):CD004568.

30. Dentali F, Crowther M, Ageno W. Thrombophilic abnormalities, oral contraceptives, and risk of cerebral vein thrombosis: a meta-analysis. Blood. 2006;107(7):2766–73.

31. Kahlenborn C, Modugno F, Potter DM, Severs WB. Oral contraceptive use as a risk factor for premenopausal breast cancer: a meta-analysis. Mayo Clin Proc. 2006;81(10):1290–302.

32. Lopez LM, Kaptein AA, Helmerhorst FM. Oral contraceptives containing drospirenone for premenstrual syndrome. Cochrane Database Syst Rev. 2012;(2):CD006586.

33. Cornish JA, Tan E, Simillis C, Clark SK, Teare J, Tekkis PP. The risk of oral contraceptives in the etiology of inflammatory bowel disease: a meta-analysis. Am J Gastroenterol. 2008;103(9):2394–400.

34. Chakhtoura Z, Canonico M, Gompel A, Thalabard J-C, Scarabin P-Y, Plu-Bureau G. Progestogen-only contraceptives and the risk of stroke: a meta-analysis. Stroke. 2009;40(4):1059–62.

35. Iodice S, Barile M, Rotmensz N, Feroce I, Bonanni B, Radice P, et al. Oral contraceptive use and breast or ovarian cancer risk in BRCA1/2 carriers: a meta-analysis. Eur J Cancer Oxf Engl 1990. 2010;46(12):2275–84.

36. Chakhtoura Z, Canonico M, Gompel A, Scarabin P-Y, Plu-Bureau G. Progestogen-only contraceptives and the risk of acute myocardial infarction: a meta-analysis. J Clin Endocrinol Metab. 2011;96(4):1169–74.

37. Manzoli L, De Vito C, Marzuillo C, Boccia A, Villari P. Oral contraceptives and venous thromboembolism: a systematic review and meta-analysis. Drug Saf. 2012;35(3):191–205.

38. Lawrie TA, Helmerhorst FM, Maitra NK, Kulier R, Bloemenkamp K, Gulmezoglu AM. Types of progestogens in combined oral contraception: effectiveness and side-effects. Cochrane Database Syst Rev. 2011;(5):CD004861.

39. Stegeman BH, de Bastos M, Rosendaal FR, van Hylckama Vlieg A, Helmerhorst FM, Stijnen T, et al. Different combined oral contraceptives and the risk of venous thrombosis: systematic review and network meta-analysis. BMJ. 2013;347(8900488, bmj, 101090866):f5298.

40. Fu Y, Zhuang Z. Long-term effects of levonorgestrel-releasing intrauterine system on tamoxifen-treated breast cancer patients: a meta-analysis. Int J Clin Exp Pathol. 2014;7(10):6419–29.

41. Soroush A, Farshchian N, Komasi S, Izadi N, Amirifard N, Shahmohammadi A. The Role of Oral Contraceptive Pills on Increased Risk of Breast Cancer in Iranian Populations: A Meta-analysis. J Cancer Prev. 2016;21(4):294–301.

© 2022 Brabaharan S et al. *JAMA Network Open*.

42. Li L, Zhong Y, Zhang H, Yu H, Huang Y, Li Z, et al. Association between oral contraceptive use as a risk factor and triple-negative breast cancer: A systematic review and meta-analysis. Mol Clin Oncol. 2017;7(1):76–80.

43. van Vlijmen EFW, Wiewel-Verschueren S, Monster TBM, Meijer K. Combined oral contraceptives, thrombophilia and the risk of venous thromboembolism: a systematic review and meta-analysis. J Thromb Haemost JTH. 2016;14(7):1393–403.

44. Ji L-W, Jing C-X, Zhuang S-L, Pan W-C, Hu X-P. Effect of age at first use of oral contraceptives on breast cancer risk: An updated meta-analysis. Medicine (Baltimore). 2019;98(36):e15719.

45. Song SY, Park M, Lee GW, Lee KH, Chang HK, Kwak SM, et al. Efficacy of levonorgestrel releasing intrauterine system as a postoperative maintenance therapy of endometriosis: A meta-analysis. Eur J Obstet Gynecol Reprod Biol. 2018;231(e4l, 0375672):85–92.

46. Roach REJ, Helmerhorst FM, Lijfering WM, Stijnen T, Algra A, Dekkers OM. Combined oral contraceptives: the risk of myocardial infarction and ischemic stroke. Cochrane Database Syst Rev. 2015;(8):CD011054.

47. Rojas-Villarraga A, Torres-Gonzalez J-V, Ruiz-Sternberg A-M. Safety of hormonal replacement therapy and oral contraceptives in systemic lupus erythematosus: a systematic review and meta-analysis. PloS One. 2014;9(8):e104303.

48. Wang A, Mo T, Li Q, Shen C, Liu M. The effectiveness of metformin, oral contraceptives, and lifestyle modification in improving the metabolism of overweight women with polycystic ovary syndrome: a network meta-analysis. Endocrine. 2019;64(2):220–32.

49. Rojas Rojas F. Second- and third-generation oral contraceptives and myocardial infarction: Systematic review and meta-analysis. Clin E Investig En Ginecol Obstet. 2016;43(4):174–8.

50. Leelakanok N, Methaneethorn J. A Systematic Review and Meta-analysis of the Adverse Effects of Levonorgestrel Emergency Oral Contraceptive. Clin Drug Investig. 2020;40(5):395–420.

51. Friebel TM, Domchek SM, Rebbeck TR. Modifiers of cancer risk in BRCA1 and BRCA2 mutation carriers: systematic review and meta-analysis. J Natl Cancer Inst. 2014;106(6):dju091.

52. Zhong G-C, Cheng J-H, Xu X-L, Wang K. Meta-analysis of oral contraceptive use and risks of all-cause and cause-specific death. Int J Gynaecol Obstet Off Organ Int Fed Gynaecol Obstet. 2015;131(3):228–33.

53. Jing Z, Liang-Zhi X, Tai-Xiang W, Ying T, Yu-Jian J. The effects of Diane-35 and metformin in treatment of polycystic ovary syndrome: an updated systematic review. Gynecol Endocrinol Off J Int Soc Gynecol Endocrinol. 2008;24(10):590–600.

54. Lethaby A, Hussain M, Rishworth JR, Rees MC. Progesterone or progestogen-releasing intrauterine systems for heavy menstrual bleeding. Cochrane Database Syst Rev. 2015;(4):CD002126.

© 2022 Brabaharan S et al. *JAMA Network Open*.

55. Chin J, Konje JC, Hickey M. Levonorgestrel intrauterine system for endometrial protection in women with breast cancer on adjuvant tamoxifen. Cochrane Database Syst Rev. 2009;(4):CD007245.

56. Amiri M, Ramezani Tehrani F, Nahidi F, Kabir A, Azizi F, Carmina E. Effects of oral contraceptives on metabolic profile in women with polycystic ovary syndrome: A meta-analysis comparing products containing cyproterone acetate with third generation progestins. Metabolism. 2017;73(mum, 0375267):22–35.

57. Lopez LM, Grimes DA, Gallo MF, Schulz KF. Skin patch and vaginal ring versus combined oral contraceptives for contraception. Cochrane Database Syst Rev. 2008;(1):CD003552.

58. Draper BH, Morroni C, Hoffman M, Smit J, Beksinska M, Hapgood J, et al. Depot medroxyprogesterone versus norethisterone oenanthate for long-acting progestogenic contraception. Cochrane Database Syst Rev. 2006;(3):CD005214.

59. Gallo MF, Nanda K, Grimes DA, Lopez LM, Schulz KF. 20 microg versus >20 microg estrogen combined oral contraceptives for contraception. Cochrane Database Syst Rev. 2008;(4):CD003989.

60. French R, Cowan F, Mansour D, Morris S, Hughes D, Robinson A, et al. Hormonally impregnated intrauterine systems (IUSs), versus other forms of reversible contraceptives as effective methods of preventing pregnancy. Cochrane Database Syst Rev. 2001;(2):CD001776.

61. Lopez LM, Grimes DA, Schulz KF. Steroidal contraceptives: effect on carbohydrate metabolism in women without diabetes mellitus. Cochrane Database Syst Rev. 2014;(4):CD006133.

62. Ralph LJ, McCoy SI, Shiu K, Padian NS. Hormonal contraceptive use and women's risk of HIV acquisition: a meta-analysis of observational studies. Lancet Infect Dis. 2015;15(2):181–9.

63. Morrison CS, Chen P-L, Kwok C, Baeten JM, Brown J, Crook AM, et al. Hormonal contraception and the risk of HIV acquisition: an individual participant data meta-analysis. PLoS Med. 2015;12(1):e1001778.

64. Gallo MF, Nanda K, Grimes DA, Lopez LM, Schulz KF. 20 micro g versus >20 micro g estrogen combined oral contraceptives for contraception. Cochrane Database Syst Rev. 2013;(8):CD003989.

65. Moorman PG, Havrilesky LJ, Gierisch JM, Coeytaux RR, Lowery WJ, Peragallo Urrutia R, et al. Oral contraceptives and risk of ovarian cancer and breast cancer among high-risk women: a systematic review and meta-analysis. J Clin Oncol Off J Am Soc Clin Oncol. 2013;31(33):4188–98.

66. Asthana S, Busa V, Labani S. Oral contraceptives use and risk of cervical cancer-A systematic review & meta-analysis. Eur J Obstet Gynecol Reprod Biol. 2020;247(e4l, 0375672):163–75.

67. Delgado-Rodriguez M, Sillero-Arenas M, Martin-Moreno JM, Galvez-Vargas R. Oral contraceptives and cancer of the cervix uteri. A meta-analysis. Acta Obstet Gynecol Scand. 1992;71(5):368–76.

68. Luan N-N, Wu L, Gong T-T, Wang Y-L, Lin B, Wu Q-J. Nonlinear reduction in risk for colorectal cancer by oral contraceptive use: a meta-analysis of epidemiological studies. Cancer Causes Control CCC. 2015;26(1):65–78.

69. Bosetti C, Bravi F, Negri E, La Vecchia C. Oral contraceptives and colorectal cancer risk: a systematic review and meta-analysis. Hum Reprod Update. 2009;15(5):489–98.

70. Xu J-L, Sun L, Liu C, Sun Z-H, Min X, Xia R. Effect of oral contraceptive use on the incidence of dry socket in females following impacted mandibular third molar extraction: a meta-analysis. Int J Oral Maxillofac Surg. 2015;44(9):1160–5.

71. Vercellini P, Eskenazi B, Consonni D, Somigliana E, Parazzini F, Abbiati A, et al. Oral contraceptives and risk of endometriosis: a systematic review and meta-analysis. Hum Reprod Update. 2011;17(2):159–70.

72. Shere M, Bapat P, Nickel C, Kapur B, Koren G. Association Between Use of Oral Contraceptives and Folate Status: A Systematic Review and Meta-Analysis. J Obstet Gynaecol Can JOGC J Obstet Gynecol Can JOGC. 2015;37(5):430–8.

73. Lan Y-L, Wang X, Lou J-C, Ma B-B, Xing J-S, Zou S, et al. Update on the effect of exogenous hormone use on glioma risk in women: a meta-analysis of case-control and cohort studies. J Neurooncol. 2018;137(2):357–65.

74. Liu H, Yao J, Wang W, Zhang D. Association between duration of oral contraceptive use and risk of hypertension: A meta-analysis. J Clin Hypertens Greenwich Conn. 2017;19(10):1032–41.

75. Ortizo R, Lee SY, Nguyen ET, Jamal MM, Bechtold MM, Nguyen DL. Exposure to oral contraceptives increases the risk for development of inflammatory bowel disease: a meta-analysis of case-controlled and cohort studies. Eur J Gastroenterol Hepatol. 2017;29(9):1064–70.

76. Liu H., Wang X.-C., Hu G.-H., Huang T.-B., Xu Y.-F. Oral contraceptive use and kidney cancer risk among women: Evidence from a meta-analysis. Int J Clin Exp Med. 2014;7(11):3954–63.

77. Halperin IJ, Kumar SS, Stroup DF, Laredo SE. The association between the combined oral contraceptive pill and insulin resistance, dysglycemia and dyslipidemia in women with polycystic ovary syndrome: a systematic review and meta-analysis of observational studies. Hum Reprod Oxf Engl. 2011;26(1):191–201.

78. Havrilesky LJ, Moorman PG, Lowery WJ, Gierisch JM, Coeytaux RR, Urrutia RP, et al. Oral contraceptive pills as primary prevention for ovarian cancer: a systematic review and meta-analysis. Obstet Gynecol. 2013;122(1):139–47.

79. Xu Z, Li Y, Tang S, Huang X, Chen T. Current use of oral contraceptives and the risk of first-ever ischemic stroke: A meta-analysis of observational studies. Thromb Res. 2015;136(1):52–60.

80. Johnston SC, Colford JMJ, Gress DR. Oral contraceptives and the risk of subarachnoid hemorrhage: a meta-analysis. Neurology. 1998;51(2):411–8.

© 2022 Brabaharan S et al. *JAMA Network Open*.

81. Perez-Lopez FR, Perez-Roncero GR, Lopez-Baena MT, Santabarbara J, Chedraui P. Hormonal contraceptives and the risk of suicide: a systematic review and meta-analysis. Eur J Obstet Gynecol Reprod Biol. 2020;251(e4l, 0375672):28–35.

82. Baratloo A, Safari S, Rouhipour A, Hashemi B, Rahmati F, Motamedi M, et al. The Risk of Venous Thromboembolism with Different Generation of Oral Contraceptives; a Systematic Review and Meta-Analysis. Emerg Tehran Iran. 2014;2(1):1–11.

83. Bateson D, Butcher BE, Donovan C, Farrell L, Kovacs G, Mezzini T, et al. Risk of venous thromboembolism in women taking the combined oral contraceptive: A systematic review and meta-analysis. Aust Fam Physician. 2016;45(1):59–64.

84. Martinez F, Ramirez I, Perez-Campos E, Latorre K, Lete I. Venous and pulmonary thromboembolism and combined hormonal contraceptives. Systematic review and meta-analysis. Eur J Contracept Reprod Health Care Off J Eur Soc Contracept. 2012;17(1):7–29.

85. Dragoman MV, Tepper NK, Fu R, Curtis KM, Chou R, Gaffield ME. A systematic review and meta-analysis of venous thrombosis risk among users of combined oral contraception. Int J Gynaecol Obstet Off Organ Int Fed Gynaecol Obstet. 2018;141(3):287–94.

86. Mantha S, Karp R, Raghavan V, Terrin N, Bauer KA, Zwicker JI. Assessing the risk of venous thromboembolic events in women taking progestin-only contraception: a meta-analysis. BMJ. 2012;345(8900488, bmj, 101090866):e4944.

87. Oedingen C, Scholz S, Razum O. Systematic review and meta-analysis of the association of combined oral contraceptives on the risk of venous thromboembolism: The role of the progestogen type and estrogen dose. Thromb Res. 2018;165(vrn, 0326377):68–78.

88. Zhu H, Lei X, Feng J, Wang Y. Oral contraceptive use and risk of breast cancer: a meta-analysis of prospective cohort studies. Eur J Contracept Reprod Health Care Off J Eur Soc Contracept. 2012;17(6):402–14.

89. Nindrea R.D., Anwar S.L., Harahap W.A., Lazuardi L., Dwiprahasto I., Aryandono T. Oral contraceptive used more than 5 years is associated with increased risk of breast cancer: A meta-analysis of 28,776 South east Asian women. Syst Rev Pharm. 2019;10(2):137–48.

90. Conz L, Mota BS, Bahamondes L, Teixeira Doria M, Francoise Mauricette Derchain S, Rieira R, et al. Levonorgestrel-releasing intrauterine system and breast cancer risk: A systematic review and meta-analysis. Acta Obstet Gynecol Scand. 2020;99(8):970–82.

91. Goshtasebi A, Subotic Brajic T, Scholes D, Beres Lederer Goldberg T, Berenson A, Prior JC. Adolescent use of combined hormonal contraception and peak bone mineral density accrual: A meta-analysis of international prospective controlled studies. Clin Endocrinol (Oxf). 2019;90(4):517–24.

92. Song J, Jin Z, Han H, Li M, Guo Y, Guo H, et al. Hormone replacement therapies, oral contraceptives, reproductive factors and colorectal adenoma risk: a systematic review and dose-response meta-analysis of observational studies. Colorectal Dis Off J Assoc Coloproctology G B Irel. 2019;21(7):748–59.

© 2022 Brabaharan S et al. *JAMA Network Open*.

93. An N. Oral Contraceptives Use and Liver Cancer Risk: A Dose-Response Meta-Analysis of Observational Studies. Medicine (Baltimore). 2015;94(43):e1619.

94. Stampfer MJ, Willett WC, Colditz GA, Speizer FE, Hennekens CH. Past use of oral contraceptives and cardiovascular disease: a meta-analysis in the context of the Nurses' Health Study. Am J Obstet Gynecol. 1990;163(1 Pt 2):285–91.

95. Peragallo Urrutia R, Coeytaux RR, McBroom AJ, Gierisch JM, Havrilesky LJ, Moorman PG, et al. Risk of acute thromboembolic events with oral contraceptive use: a systematic review and meta-analysis. Obstet Gynecol. 2013;122(2 Pt 1):380–9.

96. Khader YS, Rice J, John L, Abueita O. Oral contraceptives use and the risk of myocardial infarction: a meta-analysis. Contraception. 2003;68(1):11–7.

97. Lagergren K, Lagergren J, Brusselaers N. Hormone replacement therapy and oral contraceptives and risk of oesophageal adenocarcinoma: a systematic review and meta-analysis. Int J Cancer. 2014;135(9):2183–90.

98. Qi S, Xin R, Guo W, Liu Y. Meta-analysis of oral contraceptives and rheumatoid arthritis risk in women. Ther Clin Risk Manag. 2014;10(101253281):915–23.

99. Chen Q, Jin Z, Xiang C, Cai Q, Shi W, He J. Absence of protective effect of oral contraceptive use on the development of rheumatoid arthritis: a meta-analysis of observational studies. Int J Rheum Dis. 2014;17(7):725–37.

100. Pladevall-Vila M, Delclos GL, Varas C, Guyer H, Brugues-Tarradellas J, Anglada-Arisa A. Controversy of oral contraceptives and risk of rheumatoid arthritis: meta-analysis of conflicting studies and review of conflicting meta-analyses with special emphasis on analysis of heterogeneity. Am J Epidemiol. 1996;144(1):1–14.

101. Xu Z, Yue Y, Bai J, Shen C, Yang J, Huang X, et al. Association between oral contraceptives and risk of hemorrhagic stroke: a meta-analysis of observational studies. Arch Gynecol Obstet. 2018;297(5):1181–91.

102. Li F, Zhu L, Zhang J, He H, Qin Y, Cheng Y, et al. Oral Contraceptive Use and Increased Risk of Stroke: A Dose-Response Meta-Analysis of Observational Studies. Front Neurol. 2019;10(101546899):993.

103. Wu L, Zhu J. Linear reduction in thyroid cancer risk by oral contraceptive use: a dose-response meta-analysis of prospective cohort studies. Hum Reprod Oxf Engl. 2015;30(9):2234–40.

104. Dayan N, Holcroft CA, Tagalakis V. The risk of venous thrombosis, including cerebral vein thrombosis, among women with thrombophilia and oral contraceptive use: a meta-analysis. Clin Appl Thromb Off J Int Acad Clin Appl Thromb. 2011;17(6):E141-52.

105. de Bastos M, Stegeman BH, Rosendaal FR, Van Hylckama Vlieg A, Helmerhorst FM, Stijnen T, et al. Combined oral contraceptives: venous thrombosis. Cochrane Database Syst Rev. 2014;(3):CD010813.

106. Glisic M, Shahzad S, Tsoli S, Chadni M, Asllanaj E, Rojas LZ, et al. Association between progestin-only contraceptive use and cardiometabolic outcomes: A systematic review and meta-analysis. Eur J Prev Cardiol. 2018;25(10):1042–52.

© 2022 Brabaharan S et al. *JAMA Network Open*.
